# Supplementary material for: The telomere-to-telomere genome of flowering cherry (Prunus campanulata) reveals genomic evolution of the subgenus Cerasus
Source: Gigascience. 2025 Feb 21;14:giaf009. doi: 10.1093/gigascience/giaf009 (PMC11843098; doi:10.1093/gigascience/giaf009)
Supplement: giaf009_Supplemental_Files [file giaf009_supplemental_files.zip › Figure S7 Contraction and Expansion.pdf]

A

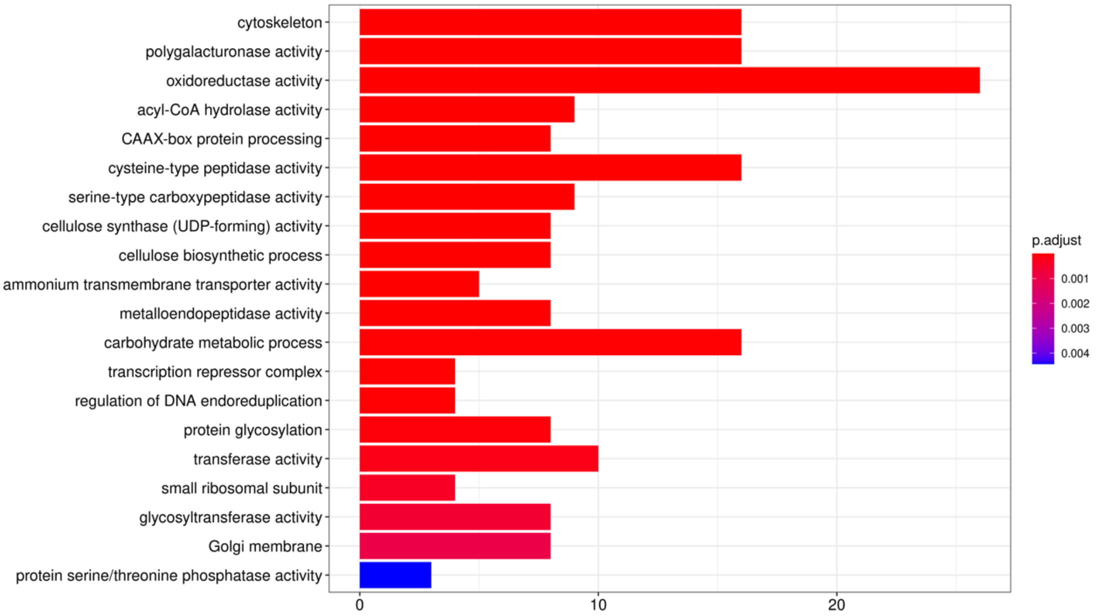

cytoskeleton
polygalacturonase activity
oxidoreductase activity
acyl-CoA hydrolase activity
CAAX-box protein processing
cysteine-type peptidase activity
serine-type carboxypeptidase activity
cellulose synthase (UDP-forming) activity
cellulose biosynthetic process
ammonium transmembrane transporter activity
metalloendopeptidase activity
carbohydrate metabolic process
transcription repressor complex
regulation of DNA endoreduplication
protein glycosylation
transferase activity
small ribosomal subunit
glycosyltransferase activity
Golgi membrane
protein serine/threonine phosphatase activity
0
10
20
p.adjust
0.001
0.002
0.003
0.004

B

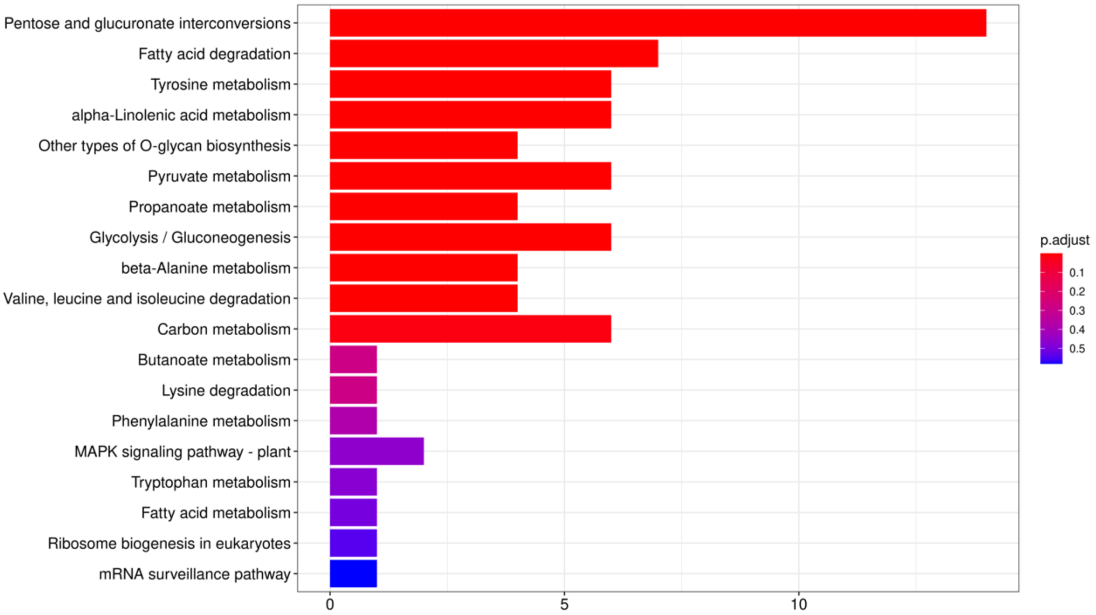

Pentose and glucuronate interconversions
Fatty acid degradation
Tyrosine metabolism
alpha-Linolenic acid metabolism
Other types of O-glycan biosynthesis
Pyruvate metabolism
Propanoate metabolism
Glycolysis / Gluconeogenesis
beta-Alanine metabolism
Valine, leucine and isoleucine degradation
Carbon metabolism
Butanoate metabolism
Lysine degradation
Phenylalanine metabolism
MAPK signaling pathway - plant
Tryptophan metabolism
Fatty acid metabolism
Ribosome biogenesis in eukaryotes
mRNA surveillance pathway
0
5
10
p.adjust
0.1
0.2
0.3
0.4
0.5

C

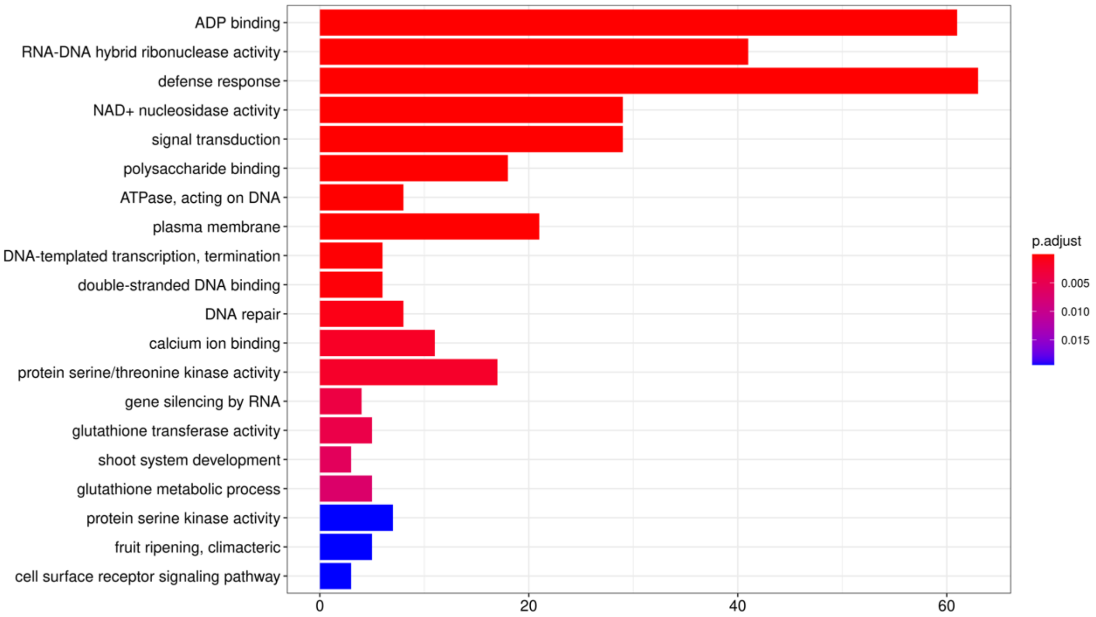

D

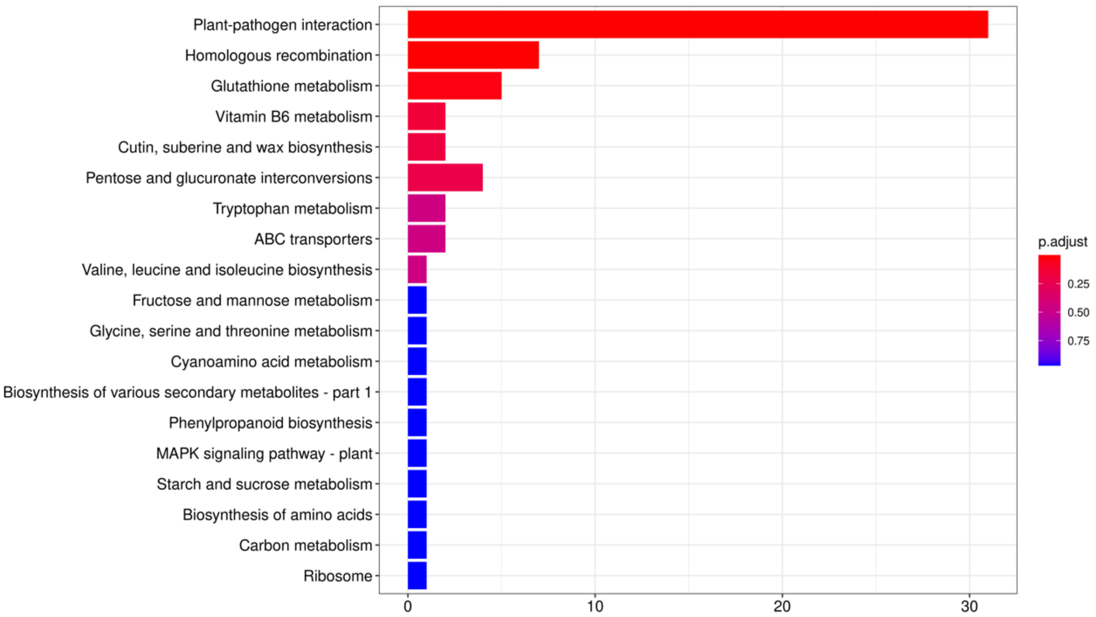

Figure S7: GO and KEGG pathway enrichment analysis of expansion and contraction gene families in P. campanulata v2.0 genome. (A) GO enrichment for expansion gene families. (B) KEGG pathway enrichment for expansion gene families. (C) GO enrichment for contraction gene families. (D) KEGG pathway enrichment for contraction gene families.
