# Supplementary figures and images for: The telomere-to-telomere genome of flowering cherry (Prunus campanulata) reveals genomic evolution of the subgenus Cerasus
Source: Gigascience. 2025 Feb 21;14:giaf009. doi: 10.1093/gigascience/giaf009 (PMC11843098; doi:10.1093/gigascience/giaf009)

## GenomeScope Profile

len:295,308,555bp uniq:50.8%  
aa:99.4% ab:0.598%  
kcov:78.3 err:0.81% dup:2.59 k:19 p:2

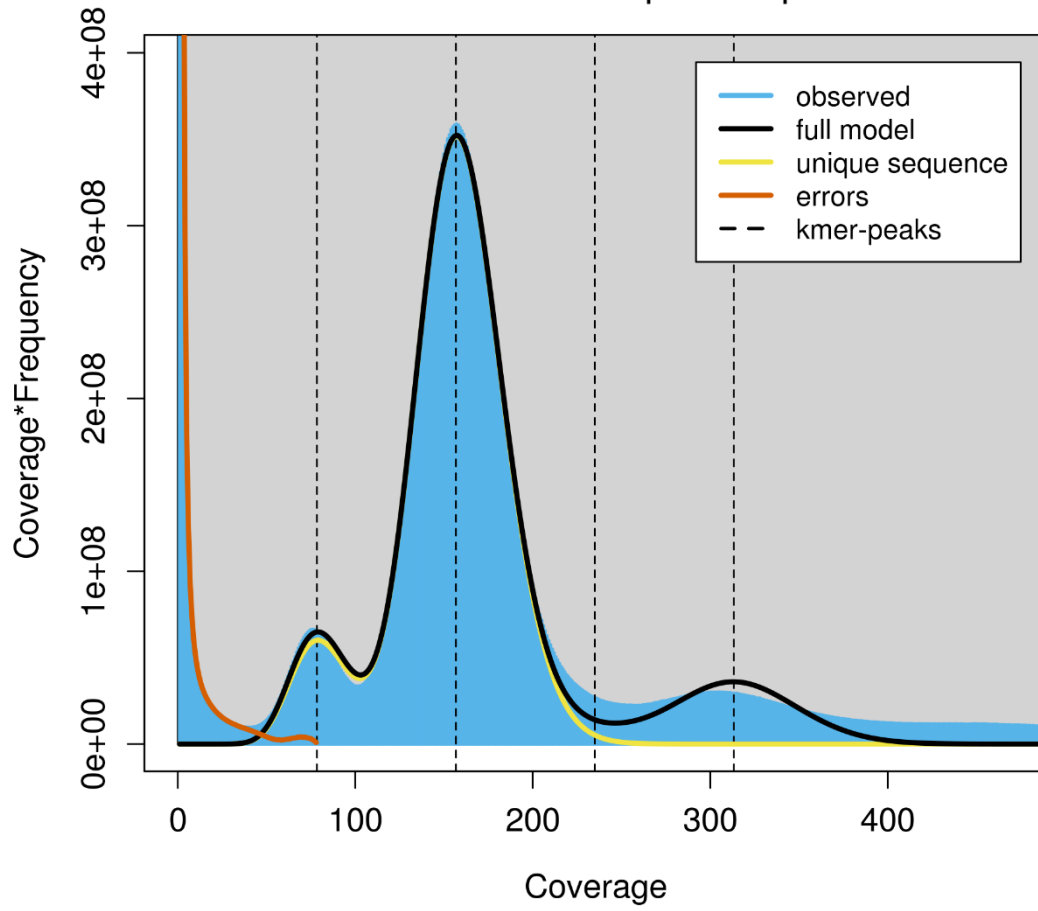

Supplement: giaf009_Supplemental_Files [file giaf009_supplemental_files.zip › Figure S1 K-mer.pdf]

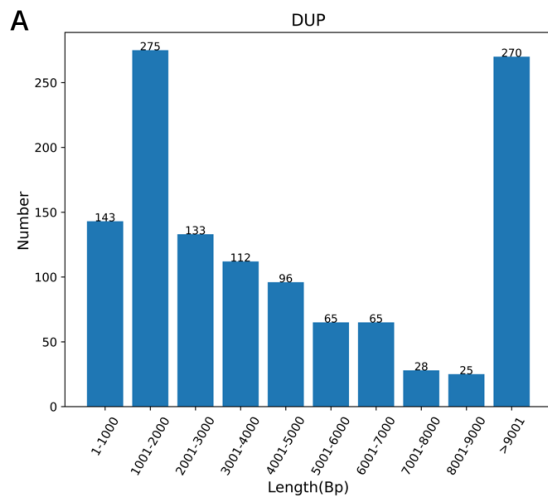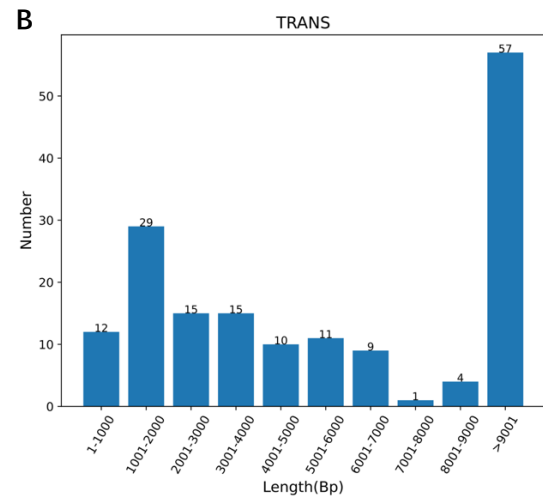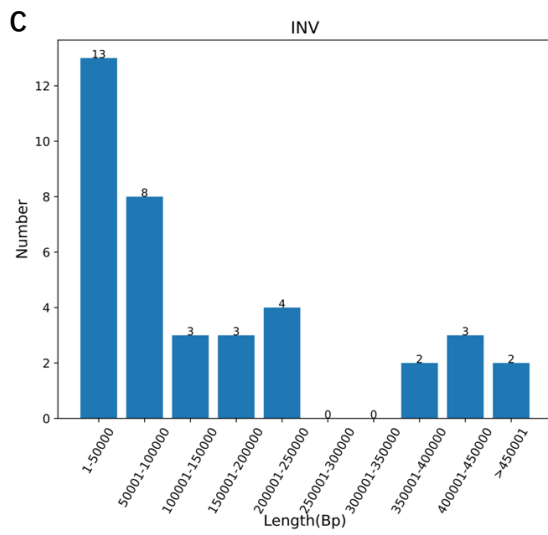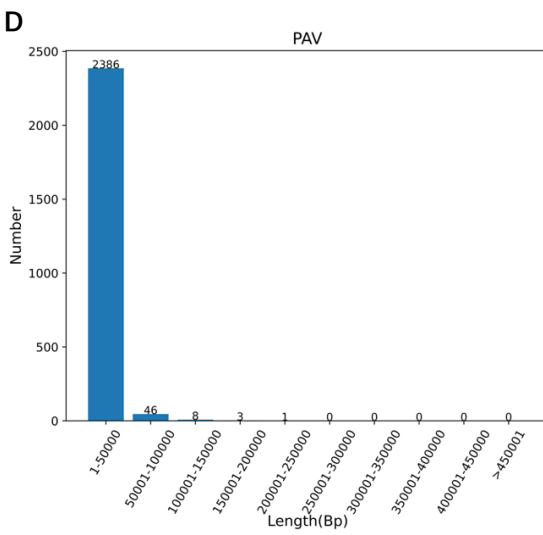

Supplement: giaf009_Supplemental_Files [file giaf009_supplemental_files.zip › Figure S2 SV.pdf]

A

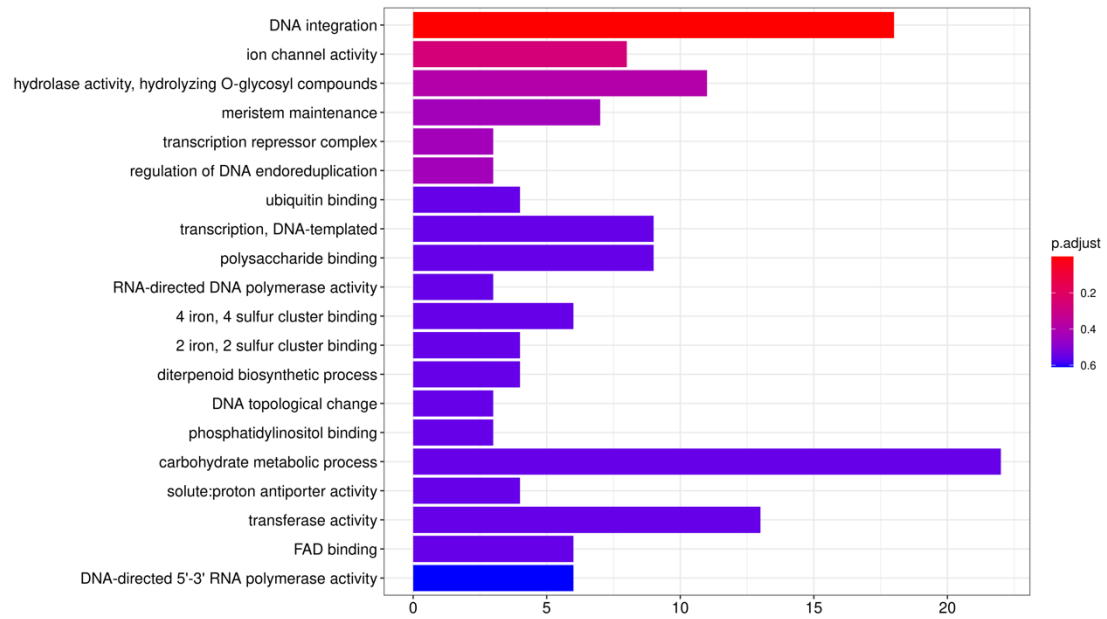

B

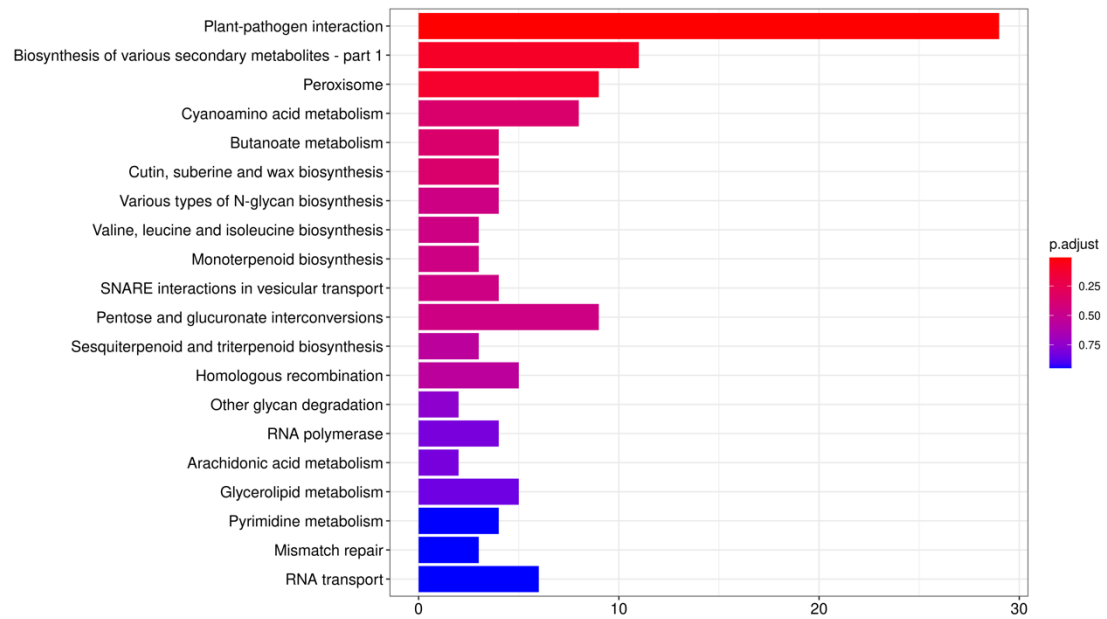

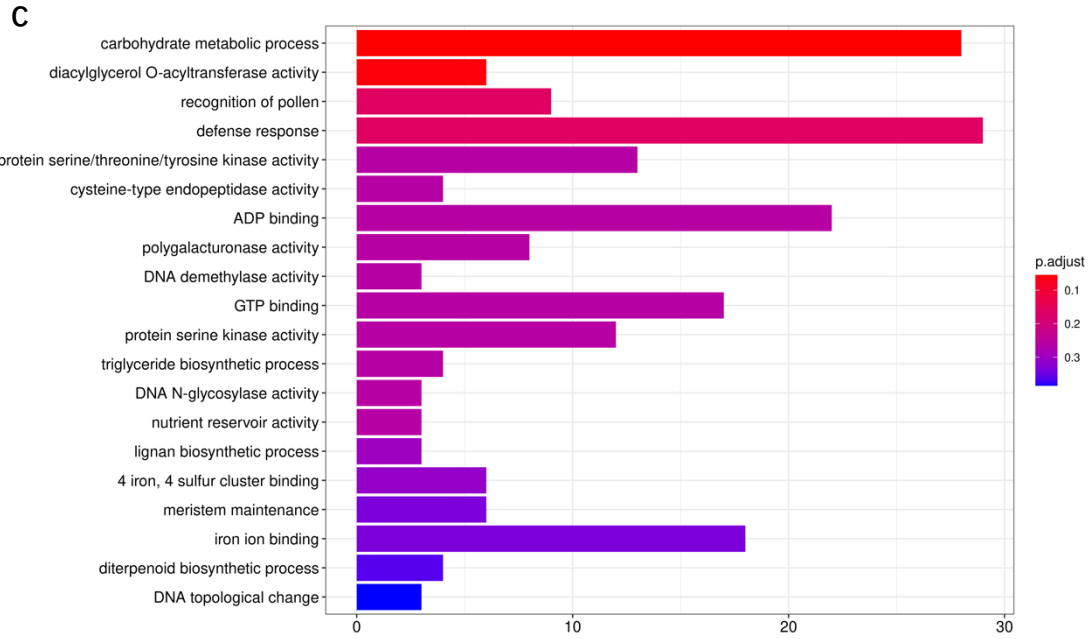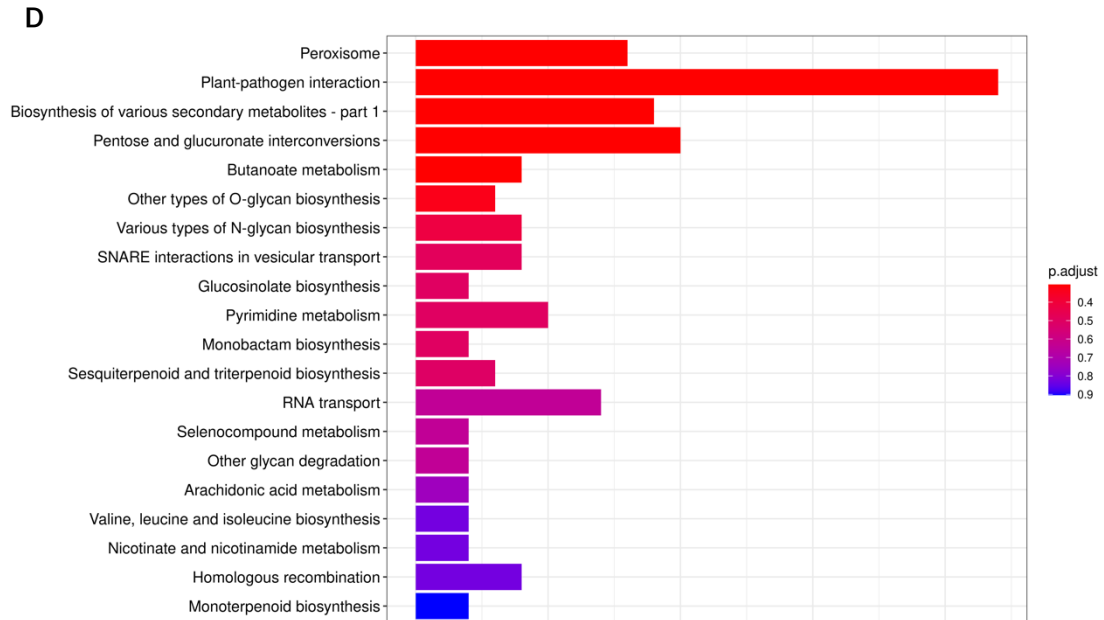

Supplement: giaf009_Supplemental_Files [file giaf009_supplemental_files.zip › Figure S3 SV go and kegg.pdf]

A

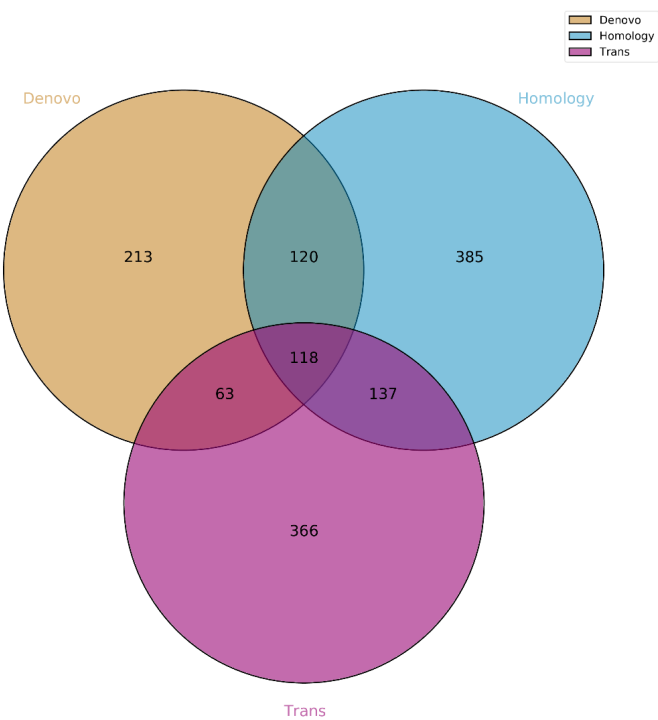

B

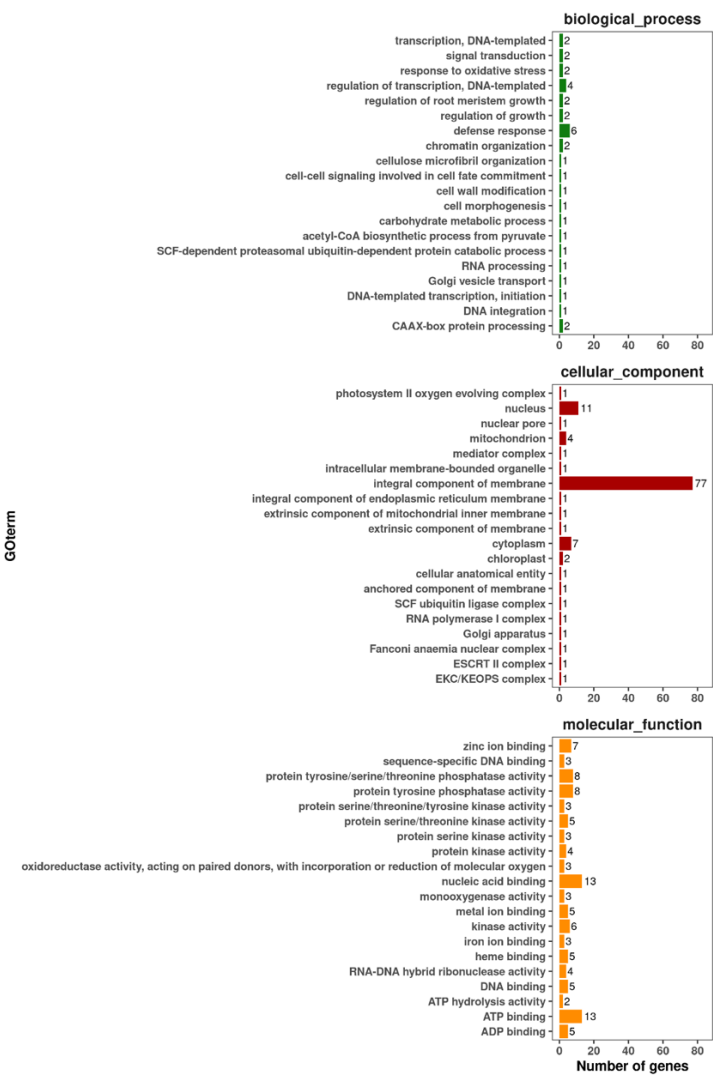

C

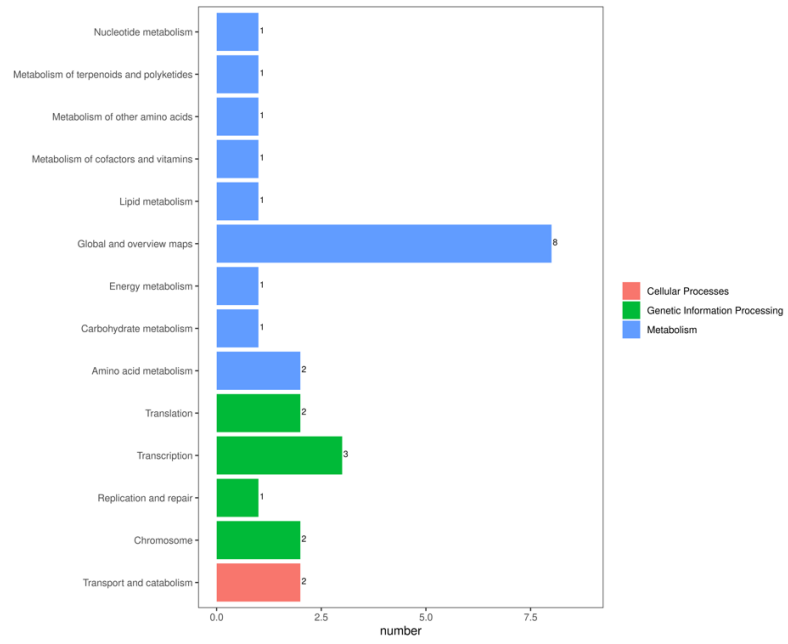

Supplement: giaf009_Supplemental_Files [file giaf009_supplemental_files.zip › Figure S4 New gene.pdf]

A

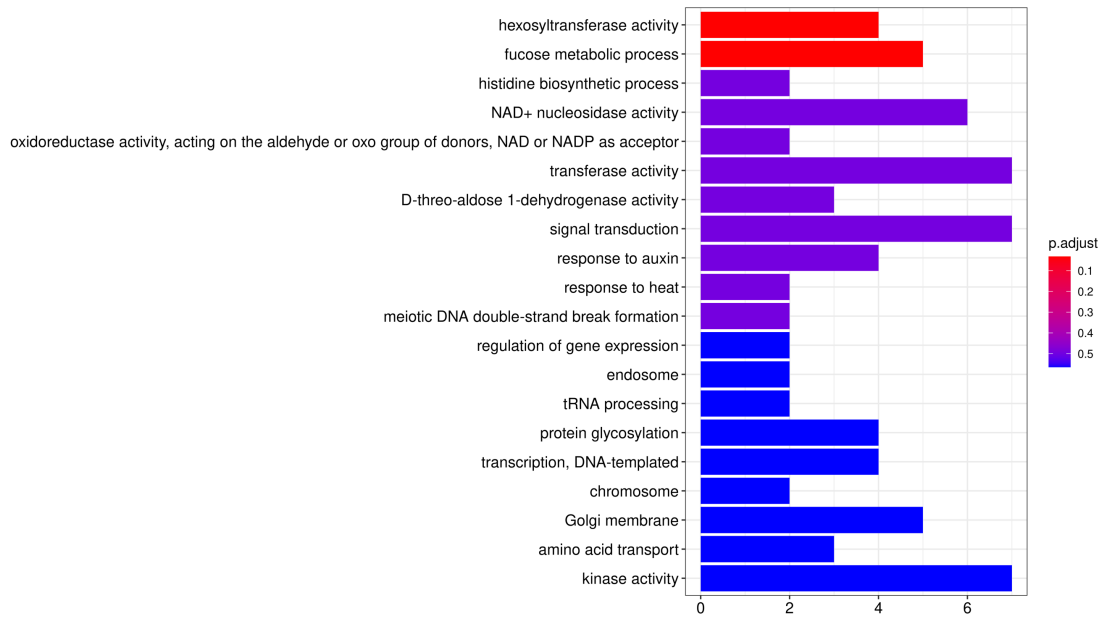

B

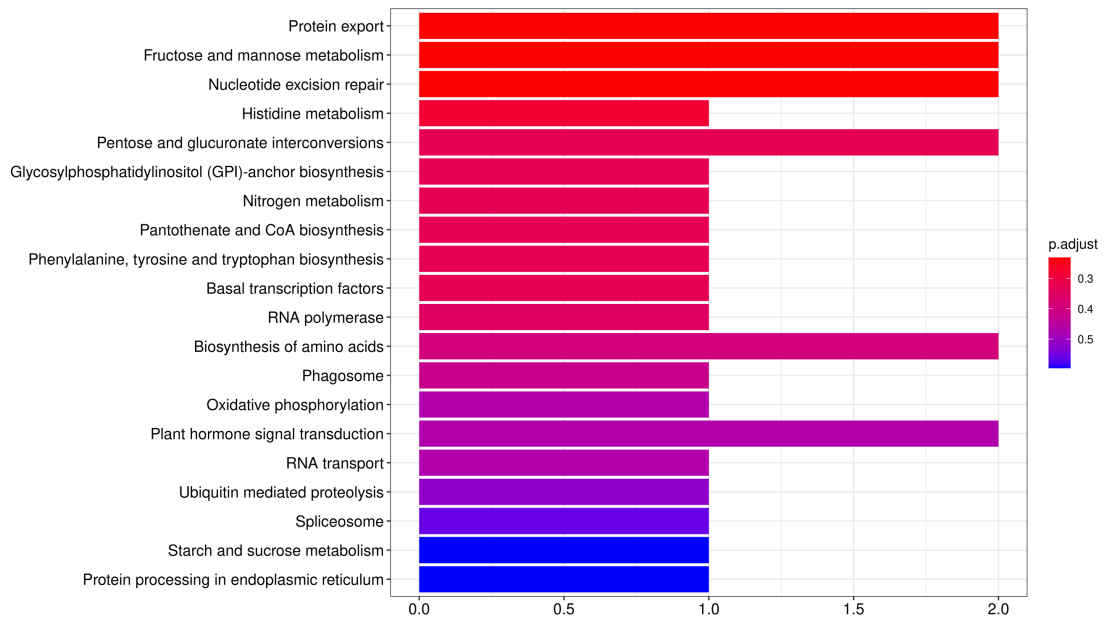

Supplement: giaf009_Supplemental_Files [file giaf009_supplemental_files.zip › Figure S5 Unique gene families.pdf]

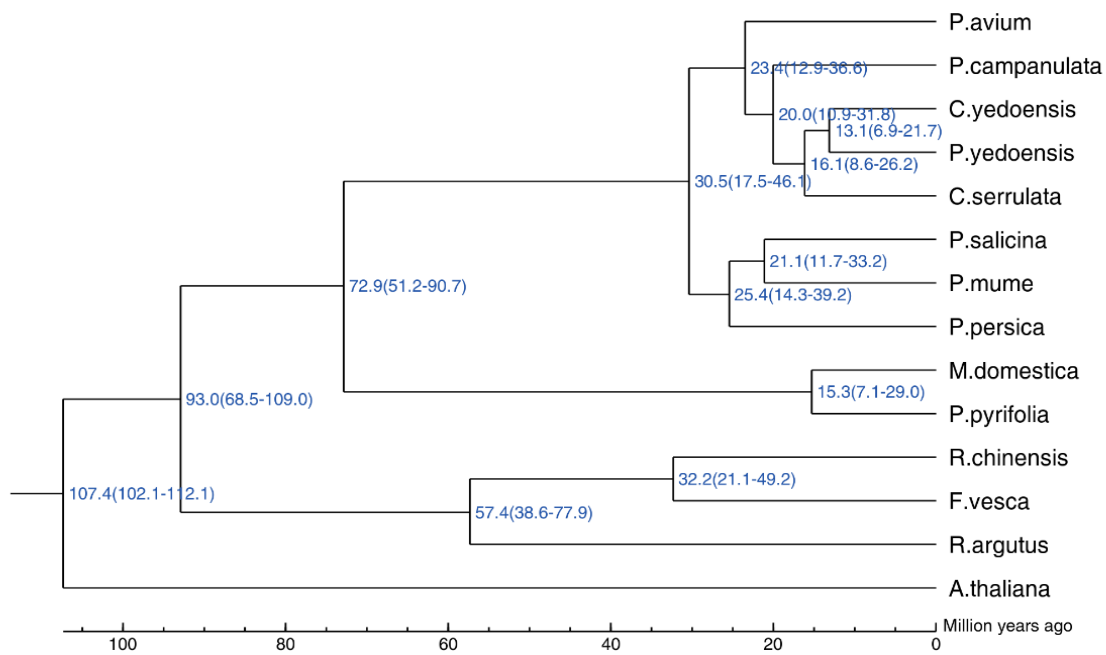

Supplement: giaf009_Supplemental_Files [file giaf009_supplemental_files.zip › Figure S6 Phylogenetic tree and divertime.pdf]

A

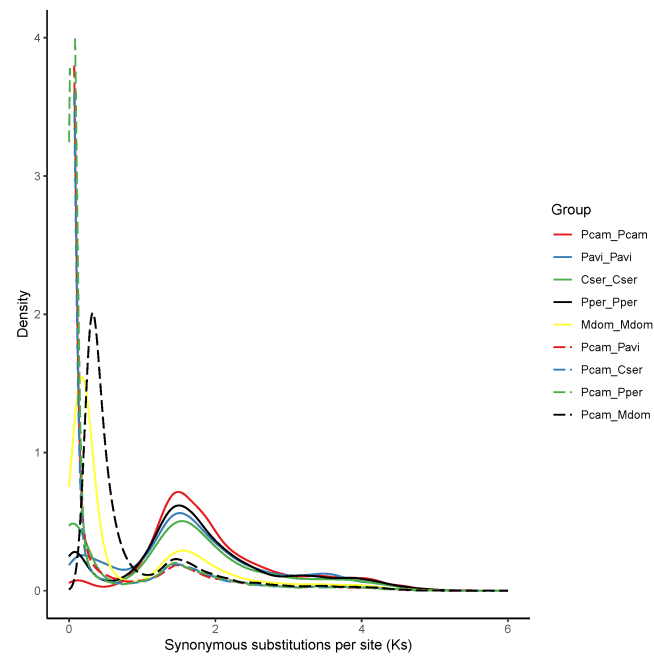

B

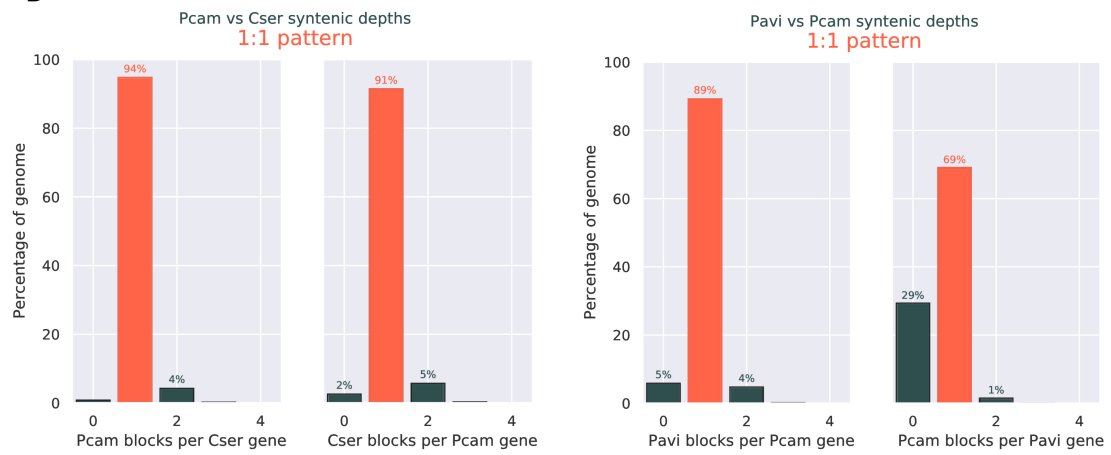

Supplement: giaf009_Supplemental_Files [file giaf009_supplemental_files.zip › Figure S8 WGD.pdf]
