# Supplementary material for: The telomere-to-telomere genome of flowering cherry (Prunus campanulata) reveals genomic evolution of the subgenus Cerasus
Source: Gigascience. 2025 Feb 21;14:giaf009. doi: 10.1093/gigascience/giaf009 (PMC11843098; doi:10.1093/gigascience/giaf009)
Supplement: giaf009_GIGA-D-24-00376_Original_Submission [file giaf009_giga-d-24-00376_original_submission.pdf]

GigaScience

The telomere-to-telomere genome of flowering cherry (Prunus campanulata) reveals genomic evolution of the subgenus Cerasus

--Manuscript Draft--

**Manuscript Number:**	GIGA-D-24-00376
**Full Title:**	The telomere-to-telomere genome of flowering cherry (Prunus campanulata) reveals genomic evolution of the subgenus Cerasus
**Article Type:**	Data Note
**Funding Information:**	Special Support Funds of Zhejiang for Scientific Research Institutes (2023F1068-2) — Dr. Fei Zhuge<br>Zhejiang Science and Technology Major Program on Agricultural New Variety Breeding (2021C02071-4) — Professor Xinhong Liu<br>National Natural Science Foundation of China (32101585) — Dr. Xin Shen
**Abstract:**	Background<br><br>Prunus campanulata, a species of ornamental cherry, holds significant genetic and horticultural value. Despite the availability of various cherry genomes, a fully resolved telomere-to-telomere (T2T) assembly for this species has been lacking. Recent advancements in long-read sequencing technologies have made it possible to generate gap-free genome assemblies, providing comprehensive insights into genomic structures that were previously inaccessible.<br><br>Findings<br><br>We present the first T2T genome assembly for P. campanulata ‘Lianmeiren’ (v2.0), achieved through the integration of PacBio HiFi, ultra-long Oxford Nanopore Technologies, Illumina, and Hi-C sequencing. The assembly resulted in a highly contiguous genome with a total size of 266.23 Mb and a contig N50 of 31.6 Mb. The genome exhibits remarkable completeness (98.9% BUSCO) and high accuracy (QV of 48.75). Additionally, 13 telomeres and putative centromere regions were successfully identified across the eight pseudochromosomes. Comparative analysis with the previous v1.0 assembly revealed 336,943 SNPs, 107,521 InDels, and 1,413 structural variations, along with the annotation of 1,402 new genes.<br><br>Conclusions<br><br>This T2T genome assembly of P. campanulata ‘Lianmeiren’ provides a critical reference for understanding the genetic architecture of the species. It enhances our ability to study structural variations, gene function, and evolutionary biology within the Prunus genus.
**Corresponding Author:**	Xin Shen<br>Zhejiang Academy of Forestry<br>Hangzhou, CHINA
**Corresponding Author Secondary Information:**	
**Corresponding Author's Institution:**	Zhejiang Academy of Forestry
**Corresponding Author's Secondary Institution:**	
**First Author:**	Dongyue Jiang
**First Author Secondary Information:**	

Powered by Editorial Manager® and ProduXion Manager® from Aries Systems Corporation

Order of Authors:	Dongyue Jiang
	Xinhong Liu
	Yingang Li
	Fei Zhuge
	Qi Zhou
	Wenjin Zong
	Xin Shen
Order of Authors Secondary Information:	
Additional Information:	
**Question**	**Response**
Are you submitting this manuscript to a special series or article collection?	No
**Experimental design and statistics**<br><br>Full details of the experimental design and statistical methods used should be given in the Methods section, as detailed in our Minimum Standards Reporting Checklist. Information essential to interpreting the data presented should be made available in the figure legends.<br><br>Have you included all the information requested in your manuscript?	Yes
**Resources**<br><br>A description of all resources used, including antibodies, cell lines, animals and software tools, with enough information to allow them to be uniquely identified, should be included in the Methods section. Authors are strongly encouraged to cite Research Resource Identifiers (RRIDs) for antibodies, model organisms and tools, where possible.<br><br>Have you included the information requested as detailed in our Minimum Standards Reporting Checklist?	Yes
**Availability of data and materials**	Yes

All datasets and code on which the conclusions of the paper rely must be either included in your submission or deposited in publicly available repositories (where available and ethically appropriate), referencing such data using a unique identifier in the references and in the "Availability of Data and Materials" section of your manuscript.<br><br>Have you have met the above requirement as detailed in our Minimum Standards Reporting Checklist?	

Powered by Editorial Manager® and ProduXion Manager® from Aries Systems Corporation

Manuscript Click here to access/download;Manuscript;manuscript-re.docx

The telomere-to-telomere genome of flowering cherry (*Prunus campanulata*) reveals genomic evolution of the subgenus *Cerasus*

Xin Shen, Xinhong Liu, Yingang Li, Fei Zhuge, Qi Zhou, Wenjin Zong and Dongyue Jiang*

Institute of Tree Breeding, Zhejiang Academy of Forestry, Hangzhou 310023, China
*Correspondence address. Dongyue Jiang, Institute of Tree Breeding, Zhejiang Academy of Forestry, Hangzhou 310023, China
E-mail: jiangdongyue@zjforestry.ac.cn

Abstract

Background: *Prunus campanulata*, a species of ornamental cherry, holds significant genetic and horticultural value. Despite the availability of various cherry genomes, a fully resolved telomere-to-telomere (T2T) assembly for this species has been lacking. Recent advancements in long-read sequencing technologies have made it possible to generate gap-free genome assemblies, providing comprehensive insights into genomic structures that were previously inaccessible.

Findings: We present the first T2T genome assembly for *P. campanulata* 'Lianmeiren' (v2.0), achieved through the integration of PacBio HiFi, ultra-long Oxford Nanopore Technologies, Illumina, and Hi-C sequencing. The assembly resulted in a highly contiguous genome with a total size of 266.23 Mb and a contig N50 of 31.6 Mb. The genome exhibits remarkable completeness (98.9% BUSCO) and high accuracy (QV of 48.75). Additionally, 13 telomeres and putative centromere regions were successfully identified across the eight pseudochromosomes. Comparative analysis with the previous v1.0 assembly revealed 336,943 SNPs, 107,521 InDels, and 1,413 structural variations, along with the annotation of 1,402 new genes.

Conclusions: This T2T genome assembly of *P. campanulata* 'Lianmeiren' provides a critical reference for understanding the genetic architecture of the species. It enhances our ability to study structural variations, gene function, and evolutionary biology within the *Prunus* genus.
Keywords: *Prunus campanulata*, T2T genome, comparative genomics, structural variations, cherry genomics

Data Description

Context

Cherry trees, known for their beautiful blossoms and abundant yield, have significant ornamental and economic value. Species of cherry belong to the subgenus *Cerasus* of *Prunus* that originated in China and is now distributed across the Northern Hemisphere [1–3]. The subgenus *Cerasus* encompasses approximately 50 to 60 species and varieties, with the majority found in China.

However, only a select few species are cultivated. One such species, *P. campanulata* Maxim., native to southern China, is highly valued as an ornamental [4, 5]. *P. campanulata* present a set of favorable traits such as early flowering, vibrant colored flowers, disease resistance, self-compatibility, and abundant seed production, making it a prime genetic resource for developing superior cultivars [6–8]. Additionally, the low ploidy level (2n = 16), small genome size, and low heterozygosity render *P. campanulata* an ideal model for studies of the cherry genome.

The advent of accurate long-read sequencing technology has brought the telomere-to-telomere (T2T) concept to the forefront of plant genomics research [9]. This approach marks a revolutionary shift in genomic sequencing and assembly, focusing on the creation of complete, continuous sequences from the end of one chromosome to another [10]. The T2T genome offers insights into the structure of centromeres and telomeres by accurately resolving repeat sequences [11–13]. The method also facilitates the annotation of additional protein-coding genes, thereby providing avenues for advances in comparative genomics and evolutionary biology, and provides precise genome sequences for applications in genetic domestication and breeding [9, 14–17]. In the future, the T2T genome is expected to become the standard reference. While the T2T genomes of many horticultural plants have been released in recent years, the T2T genome of cherries remains unsequenced.

At present, the genomes of several cherry species and varieties have been sequenced using next-generation or third-generation sequencing platforms. These species include *P. avium* [18, 19], *P. yedoensis* [20], *C.* × *yedoensis* [21], *C. serrulata* [22], *C.* × *kanzakura* [23], *P. fruticosa* [24], *P. campanulata* [4, 5], *P. pusilliflora* [25], *P. cerasus* [26], and *P. conradinae* [27]. In February 2023, we reported a chromosome-level assembly of *P. campanulata* 'Lianmeiren' (v1.0) that was achieved using a combination of PacBio, 10x Genomics, and Illumina sequencing technologies [4]. This was closely followed by the publication of another chromosome-scale genome of *P. campanulata* 'Plena' [5]. Despite these advancements, challenges remain in resolving gaps and highly repetitive regions within the cherry genome, highlighting the need for ongoing refinement and improvement.

To tackle the existing challenges, we have assembled a high-quality T2T genome of *P. campanulata*. This assembly was achieved through the integration of ultra-long ONT, PacBio HiFi, Illumina, and Hi-C sequencing. The completion of a gap-free *P. campanulata* genome significantly advances our understanding of the cherry genome and paves the way for new opportunities in the utilization of cherry germplasm resources.

Methods

Plant materials and sequencing

P. campanulata 'Lianmeiren', a double-flowered cherry variety, was cultivated at the Zhejiang Academy of Forestry nursery in Hangzhou, China (Fig. 1A). During the fruit-ripening stage, fresh young leaves were harvested and immediately preserved in liquid nitrogen for DNA extraction. We employed the CTAB method to prepare high-molecular-weight genomic DNA that was subsequently purified using a Qiagen genomic kit (Qiagen, 13343) for PacBio HiFi sequencing.

Additionally, DNA for ultra-long Oxford Nanopore Technologies (ONT) sequencing was extracted via the SDS method. The quality of the DNA was assessed using a NanoDrop One spectrophotometer (NanoDrop Technologies, Wilmington, DE, USA) and a Qubit 3.0 Fluorometer (Life Technologies, Carlsbad, CA, USA). An Ultra-Long DNA Sequencing Kit v14 (SQK-LSK114, ONT, UK) was used to create the ONT sequencing library, and a SMRTbell express template prep kit 2.0 (Pacific Biosciences, CA, USA) was employed for the preparation of the PacBio HiFi sequencing library. The sequencing of the ONT and PacBio libraries was performed on a Nanopore PromethION sequencer and the PacBio Sequel II platform, respectively. Previous studies provided Illumina and Hi-C reads for supplementary analysis.

Genome assembly and assessment

After discarding reads with Q-scores less than 7, we processed the ONT ultra-long reads by removing adapters and filtering out short reads (less than 10 kb) using Filtlong v0.2.4 (RRID: SCR_024020) and Porechop v0.2.4 (RRID: SCR_016967), respectively. We retained high-quality reads that were at least 30 kb in length and had a Q-score above 9 for further analysis. The quality of the raw HiFi reads was evaluated using CCS v6.0.0 (RRID: SCR_024379); this step involved filtering out sequences with fewer than three rounds of sequencing and low-quality subreads with a SNR below 2.5, thereby ensuring the validity of the data for subsequent analyses. Similarly, raw Illumina reads were processed with FastQC v0.21.0 (RRID: SCR_014583) [28] to eliminate low-quality reads. To facilitate chromosome-level assembly of the Hi-C data, only valid interaction pairs were extracted using HiCUP v0.8.0 (RRID: SCR_005569) [29].

Preliminary assembly of the ONT ultra-long sequencing data was conducted using NextDenovo v2.5.0 (RRID: SCR_025033) [30] with the read_cutoff set to 1k, blocksize at 1g, and nextgraph_options configured as −a1. To polish the assemblies, we utilized two iterative rounds of Racon v1.4.11 (RRID: SCR_017642) and Pilon v1.23 (RRID: SCR_014731) [31] for the ONT and Illumina reads, respectively, adhering to their default settings. For the PacBio HiFi draft genome assembly, two distinct approaches were adopted: one using Hifiasm v0.16.1-r375 (RRID: SCR_021069) [32] exclusively for PacBio HiFi data, and the other combining ONT ultra-long with PacBio HiFi data via Hifiasm v0.18.2-r467 (RRID: SCR_021069) [32]. This process yielded 52 contigs from the ONT genome assembly, 354 from the PacBio assembly, and 249 from the hybrid assembly. To screen for and remove non-target sequences, Minimap2 v2.17-r941 (RRID: SCR_018550) [33] was used to identify mitochondrial and chloroplast data. Sequences with a base alignment of over 50% were excluded. Additionally, bacterial contamination was identified and eliminated by comparing the sequences to those in the RefSeq library (RRID: SCR_003496) [34], and contigs with insufficient read support were discarded.

ALLHiC v0.9.8 (RRID: SCR_022750) [35] was employed for clustering the contig sequences into distinct chromosome groups utilizing a bottom-up hierarchical clustering algorithm. This was followed by ordering and orienting the contigs within each chromosome group. The pairwise interactions between contigs were then transformed into ".hic" files using 3D-DNA v180419 (RRID: SCR_017227) [36] and Juicer v1.6 (RRID: SCR_017226) [37]. Juicebox v1.11.08 (RRID: SCR_021172) [38] was used for manual ordering and orientation. The gap-free ONT genome

sequence was used as a reference to fill in the gaps in the genome assembled by Hifiasm v0.18.2-r467 (RRID: SCR_021069) [32]. The heterozygous sequences identified through the pairwise interaction of contigs were removed, and gaps were filled with a sequence of 100 Ns to construct the final chromosome-level genome sequence. Finally, to visualize the genomic interactions, a heat map was generated using HiCExplorer v3.6 (RRID: SCR_022111) [39].

The ONT ultra-long reads were mapped to the genome assembly using Winnowmap v1.11 (RRID: SCR_025349) [40], focusing on collecting reads at the terminals of each pseudochromosome within a 50-bp screening window. We searched for the numbers of telomere repeats (CCCATTT at the 5′ end and TTTAGGG at the 3′ end) across all reads. The read with the highest count of these repeats was designated as “ref” (reference), and the others as “query.” Both reference and query sequences were then reassembled to obtain consensus sequences using medaka_consensus v1.2.1 (https://github.com/nanoporetech/medaka). The consensus sequences were then used to replace the terminal sequences on each pseudochromosome, a process carried out using MUMmer’s nucmer v3.1 (RRID: SCR_018171) [41]. For gap filling, we compared the data and genome gap intervals, prioritizing gap filling with the sequence hierarchy of “genome version after error correction > HiFi data > ONT Ultra-long data.” Sequences that spanned the entire gap on the alignment were chosen, with preference being given to the best-aligned sequence that covered the longest length of the region. This sequence was then used to fill the gap in the genome.

For error correction, HiFi reads with a length of at least 10 kb were aligned to the gapped version of the genome using Winnowmap2 (RRID: SCR_025349) [40]. The alignment parameters included k = 15, greater-than, distinct = 0.9998, —MD, and -ax map-pb. The aligned fragments underwent filtering through SAMtools v1.10 (RRID: SCR_002105) [42], using the parameter -F 256. To remove chimeric alignments, we applied falconc bam-filter-clipped, setting parameters -t and -F 0x104. Utilizing the information from these filtered alignments, a specialized branch of Racon v1.6.0 (RRID: SCR_017642) was employed for error correction.

The continuity of the genome was evaluated by identifying the location and number of gaps in the assembly. To estimate the genome consensus, we mapped Illumina and Hi-C reads to the final assembly using BWA v0.7 (RRID: SCR_010910) [43]. Additionally, ONT and PacBio HiFi reads were aligned with Minimap2 v2.17-r941 (RRID: SCR_018550) [33]. The completeness of the genome assembly was assessed using BUSCO v5.3.0 (RRID: SCR_015008) [44]. To evaluate the quality and accuracy of the genome assembly, we compared the k-mer spectrum of Illumina sequencing reads with the assembled genome. This comparison was expressed through the consensus QV, providing a measure of assembly accuracy.

Genome annotation

To identify and classify repeat sequences, we initially utilized RepeatModeler v1.0.11 (RRID: SCR_015027) (https://www.repeatmasker.org/RepeatModeler/) for de novo prediction. This was complemented by the use of LTR_Finder (RRID: SCR_015247) [45] and LTR_retriever (RRID: SCR_017623) [46] to identify non-redundant long terminal repeat (LTR) sequences. We then combined these sequences to form a de novo repeat sequence library. This library was merged with

the Repbase v20181026 library (RRID: SCR_021169) [47] to obtain a comprehensive database. This combined library was then used in RepeatMasker v4.0.9 (RRID: SCR_012954) to predict repeat sequences throughout the genome. Additionally, RepeatProteinMask v4.0.9 (https://www.repeatmasker.org/) was used specifically for the prediction of transposable element (TE) proteins. We compiled the final set of repeat sequences after the removal of redundant sequences.

For the prediction of gene structure, we employed a combined strategy encompassing ab initio, homology-based, and RNA-seq-based methods. For ab initio prediction, we used Augustus v3.3.2 (RRID: SCR_008417) [48] and GlimmerHMM v3.0.4 (RRID: SCR_002654) [49], focusing on the genomic regions masked for repeat sequences. BUSCO v5.2.2 (RRID: SCR_015008) [44] was then used to derive training sets for this purpose. In the homology-based approach, protein sequences of *P. avium* [18], *C. serrulata* [22], *P. mume* [50], *P. persica* [51], and *P. campanulata* (v1.0) [4] were mapped to the reference genome using TBLASTN v2.7.1 (RRID: SCR_011822). This was followed by the use of Exonerate v2.4.0 (RRID: SCR_016088) [52] to predict transcripts and coding regions. For RNA-seq-based prediction, RNA-seq reads, filtered using fastp v0.21.0 (RRID: SCR_016962) [28], were initially aligned to the genome via HISAT2 v2.1.0 (RRID: SCR_015530) [53]. The resulting alignment data were then utilized to acquire transcripts with StringTie v2.1.4 (RRID: SCR_016323) [54], and these transcripts aided in predicting gene models using TransDecoder v5.1.0 (RRID: SCR_017647). Finally, we integrated all data to form the final set of gene models via MAKER v2.31.10 (RRID: SCR_005309) [55].

Gene functions were predicted based on sequence and motif similarities. This involved comparing their protein sequences against several databases: UniProt (RRID: SCR_002380) [56], Nr [57], GO (RRID: SCR_002811) [58], KOG [59], Pfam (RRID: SCR_004726) [60], InterPro (RRID: SCR_006695) [61], and KEGG (RRID: SCR_012773) [62]. For the KEGG annotations, we utilized DIAMOND v0.9.30 (RRID: SCR_016071) [63] and KOBAS v3.0 (RRID: SCR_006350) [64]. Protein domain and GO term annotations were derived using InterProScan v5.52-86.0 (RRID: SCR_005829) [65], while protein family annotations were obtained by searching the Pfam database (RRID: SCR_004726) [60] with hmmscan v3.3.2 [66]. In addition, tRNAs in the genome were identified with tRNAscan-SE v1.23 (RRID: SCR_008637) [67], focusing on their structural characteristics. rRNAs were predicted using the rRNA database, and snRNA and miRNA sequences were annotated based on the Rfam database (RRID: SCR_007891) [68] using Infernal v1.1.2 (RRID: SCR_011809) [69].

Genomic comparison between v2.0 and v1.0 assemblies

The complete T2T genome assembly was aligned pairwise with the v1.0 genome using SyRI v1.63 (RRID: SCR_023008) [70], enabling us to identify syntenic regions and various structural variants (SVs), including inversions, translocations, and duplications. For visual comparison between the T2T and v1.0 genomes, we employed OrthoVenn2 (RRID: SCR_022504) [71] to create a Venn diagram, setting an e-value threshold of 1e−10. To annotate genes newly identified in the T2T assembly, GO analysis was conducted using InterProScan (RRID: SCR_005829) [65]. This analysis focused on characterizing gene functions across biological process, cellular component, and

molecular function terms, as defined by the GO knowledgebase (RRID: SCR_017505) [58]. The R package clusterProfiler (RRID: SCR_016884) [72] was then used to perform the GO enrichment analysis and to visualize the results. Additionally, we utilized JCVI v0.9.13 [73] to identify genes that were newly annotated in the T2T genome relative to v1.0, particularly those located in inversions, duplications, and translocations.

Identification of telomere and centromere

To identify telomeres, all ONT reads were first aligned to the reference genome using Winnowmap v1.11 (RRID: SCR_025349) (parameters: k=15, –MD) [39], specifically targeting reads that aligned singularly within 50 bp of chromosomal ends. We then calculated the frequency of telomere repeat sequences ("CCCTAAA"/"TTTAGGG") in each read, referencing the Telomere database (https://telomerase.asu.edu/sequences-telomere) [74]. The read with the most telomere repeats was designated as the reference, and the others as queries. Following this, medaka_consensus v1.2.1, (parameters: -m r941_min_high_g360; https://github.com/nanoporetech/medaka) was employed to reassemble the reference and query the telomere reads, yielding a consensus sequence. This consensus sequence was then aligned to each chromosome using MUMmer's nucmer v3.1 (RRID: SCR_018171) [41], to replace terminal telomere sequences using the best alignment results. However, replacement was not conducted if the identity fell below an 80% threshold, or if the aligned region was not within 20 kb of the chromosomal end. Leveraging the distinct features of high-density short tandem repeat finders (TRFs) and low-density gene distribution in centromere regions, we employed BEDTools (RRID: SCR_006646) [75] to compute TRF and gene coverage, utilizing a 10-bp window. This analysis led to the prediction of eight centromeric regions within the chromosomes of the *P. campanulata* genome.

Evolutionary analysis

In addition to *P. campanulata*, we included 13 other plant species— *Arabidopsis thaliana* [76], *P. persica* [51], *P. mume* [50], *P. avium* [18], *C. serrulata* [22], *C. × yedoensis* [21], *P. yedoensis* [20], *P. salicina* [77], *Malus domestica* [78], *Pyrus pyrifolia* [79], *Fragaria vesca* [80], *Rubus argutus* [81], and *Rosa chinensis* [82]— for gene family clustering. This clustering was performed using BLASTP v2.6.0 (RRID: SCR_001010) [83] and OrthoFinder v2.3.12 (RRID: SCR_017118) [84]. After clustering, the R package clusterProfiler (RRID: SCR_016884) [72] was employed to conduct GO and KEGG analyses. For the analysis of single-copy orthologous gene families, protein sequences were aligned using MUSCLE v3.8.31 (RRID: SCR_011812) [85]. The alignment results were then refined with trimAl v1.2rev59 (RRID: SCR_017334) [86] and amalgamated to create a comprehensive super-alignment matrix.

Using the super-alignment matrix, we constructed a maximum likelihood (ML) phylogenetic tree with RAxML v8.2.10 (RRID: SCR_006086) [87] using the GTRGAMMA substitution model.

MCMCTree from PAML v4.9 (RRID: SCR_014932) [88] was used to estimate divergence times. We incorporated three calibration priors from TimeTree (RRID: SCR_021162) [89] in our analysis. These included divergence time estimates between *P. campanulata* and *A. thaliana* (102.0–112.5 Mya), *M. domestica* and *P. pyrifolia* (2.30–54.83 Mya), and *R. chinensis* and *F. vesca* (21.12–57.76 Mya).

Using the insights gained from the phylogenetic tree and gene family clustering, gene family expansions and contractions were identified using CAFE v3.1 [90]. To further characterize the genetic variation, clusterProfiler (RRID: SCR_016884) [72] was employed to conduct GO and KEGG enrichment analyses, providing a deeper understanding of the functional implications of the gene family dynamics.

To detect whole genome duplication (WGD) events, our initial step involved aligning the protein sequences from *P. campanulata* with those of other related species using BLAST v2.6.0+ [83]. This was followed by identifying collinear segments both within *P. campanulata* and between *P. campanulata* and related species (*P. avium*, *P. mume*, and *P. persica*) using MCScanX v0.8 (RRID: SCR_022067) [91] under the default settings. The frequency of synonymous (Ks) and non-synonymous (Ka) mutations, as well as their ratio (Ka/Ks), in these collinear gene pairs was calculated using the yn00 module of PAML v4.9 (RRID: SCR_014932) [88]. The resulting data were then visually represented in a density map created using ggplot2 v2.2.1 (RRID: SCR_014601) [92].

For the analysis of positive selection, we utilized the CodeML module in PAML v4.9 (RRID: SCR_014932) [88]. MAFFT (RRID: SCR_011811) [93] was initially employed to align protein sequences from single-copy gene families among the selected species. The protein sequences were then converted into codon sequences using PAL2NAL v14 [94]. Then CodeML using the Branch-site model was used to perform likelihood ratio tests between Model A (which assumes that the foreground branches ω are under positive selection, i.e., $\omega > 1$) and the null model (where no site is permitted to have an ω value greater than 1). These tests were conducted using the chi2 program. Genes exhibiting significant differences (p-value < 0.05) were classified as being subject to positive selection.

To identify similar gene pairs, we utilized LAST v1170 (RRID: SCR_006119) [95] to compare gene sequences between two species. Following this, JCVI v0.9.13 [73] was employed to ascertain the chromosomal positions of these similar gene pairs. We then plotted a collinear map to visually represent the relationships and alignments of these gene pairs across chromosomes.

Results

T2T assembly of *P. campanulata* genome

The initial genome survey utilizing Illumina reads estimated the genome size of *P. campanulata* ‘Lianmeiren’ to be approximately 295.31 Mb, with a heterozygosity rate of 0.60% (Supplementary

Fig. S1). To construct a T2T gap-free genome assembly of *P. campanulata*, we generated approximately 16.19 Gb (~54× coverage) of ultra-long sequencing reads using the ONT platform, and approximately 30.54 Gb (~108× coverage) of PacBio HiFi reads using the PacBio Sequel II platform (Supplementary Table S1). The N50 lengths for the HiFi and ONT ultra-long reads exceeded 15.58 kb and 100 kb, respectively (Supplementary Table S1). Additionally, around 63.83 Gb (~209× coverage) of Illumina paired-end sequencing data were utilized to correct the genome assembly and for QV evaluation. Three draft assemblies were created using ONT ultra-long reads, PacBio HiFi reads, and a hybrid assembly combining both. The assembly utilizing PacBio HiFi reads exhibited superior performance, resulting in 354 highly continuous contigs, with a contig N50 of 29.86 Mb, a QV of 53.8, and 98.9% completeness (Supplementary Table S2). This assembly was selected as the T2T genome framework. Following the exclusion of non-nuclear and contaminated sequences and contigs with low support, anchoring of contigs was performed using approximately 35.34 Gb (~117× coverage) of Hi-C sequencing data (Supplementary Table S1), organizing all contigs into eight pseudochromosomes (Fig. 1B). A complete, gap-free reference genome (v2.0) was subsequently produced by filling all remaining gaps with the gap-free ONT genome data. Most of the gaps were located in chromosomes 1, 4, 5, and 7, with sequence lengths ranging from 99 to 95,957 bp (Supplementary Table S3). The finalized genome size was 266.23 Mb, a value that was slightly lower than the estimate derived from flow cytometry (~295 Mb) (Supplementary Fig. S1), with a contig N50 of 31.6 Mb (Fig. 1C, Table 1, Supplementary Table S4).

Table 1: Genomic statistics of *P. campanulata* v2.0 assembly and previous assemblies.

**Feature**	**'Lianmeiren' v2.0**	**'Lianmeiren' v1.0**	**'Plena'**
Genome size (Mb)	266.23	299.15	280.20
Contig N50 (Mb)	31.6	2.02	18.31
Number of contigs	8	687	41
Gaps	0	/	/
Number of telomeres	13	0	0
Number of centromeres	8	0	0
Number of gene models	28,961	28,319	27,181
BUSCOs (%)	98.90	96.60	98.70

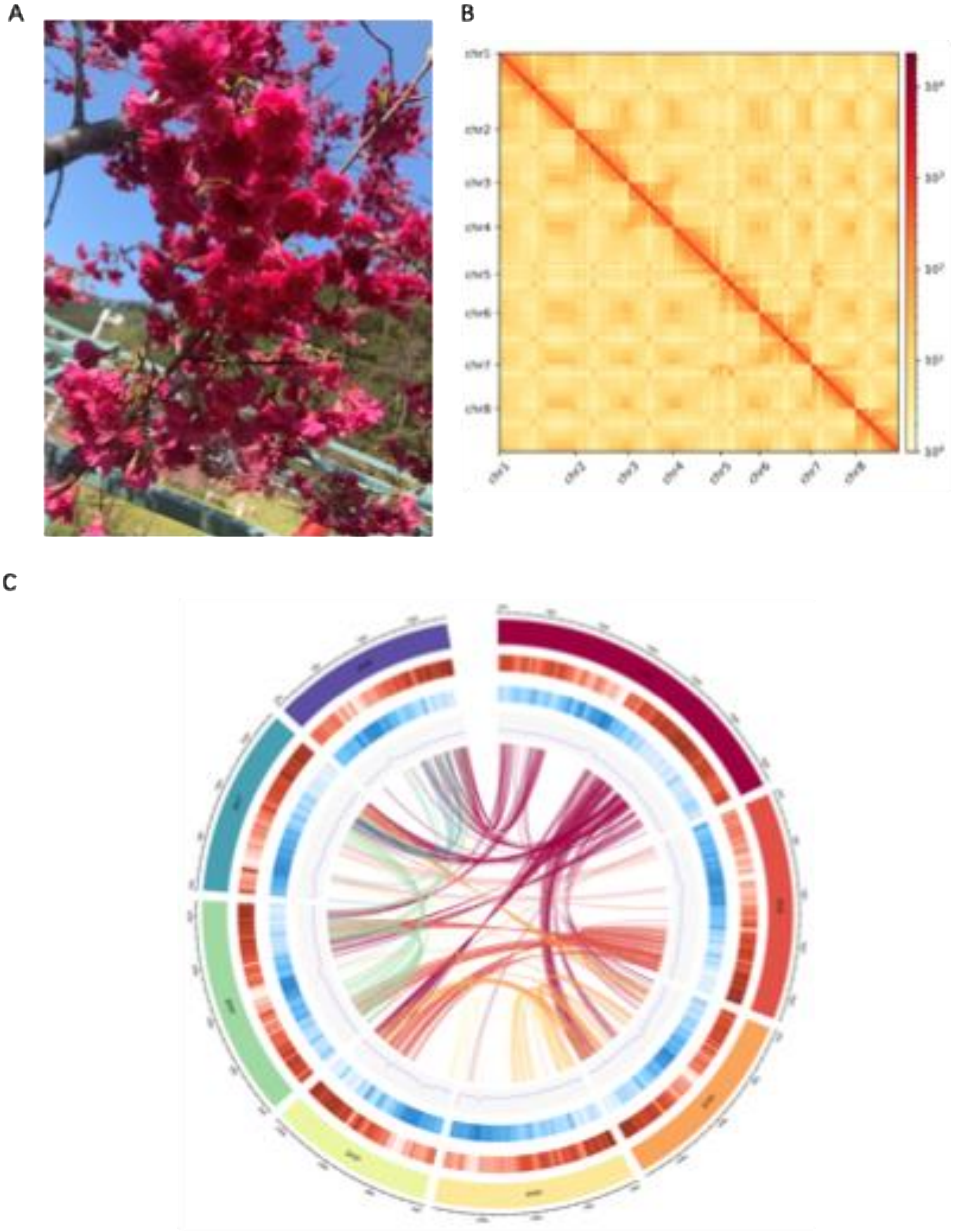

Figure 1: *P. campanulata* ‘Lianmeiren’ morphology and telomere-to-telomere (T2T) genome assembly. (A) Floral morphology of *P. campanulata* ‘Lianmeiren’. Scale bar corresponds to 1 cm. (B) Hi-C interaction heat map for eight pseudochromosomes of the *P. campanulata* genome. (C) Circos plot of the *P. campanulata* genome assembly. From outer to inner ring: chromosomes, gene density, repeat sequence density, GC Content, and gene collinearity, with a window length of 500K.

Extensive evaluation of the T2T P. campanulata assembly

The quality of the *P. campanulata* genome was assessed across four dimensions: consistency, contiguity, completeness, and accuracy. The assembly demonstrated near-perfect consistency, as

evidenced by the absence of gaps and mismatches (N) across all chromosomes, and the count of contigs precisely matched the number of chromosomes. For contiguity, 96.1% of the Illumina short reads, 99.11% of the ONT ultra-long reads, and 99.98% of the HiFi reads could be aligned to the assemblies, covering 100%, 99.99%, and 99.99% of the respective assembly regions (Supplementary Table S5). Completeness was evaluated using BUSCO, with 98.9% (N = 1,614) of conserved plant genes identified as complete (Supplementary Table S6). The k-mer statistical analysis indicated a QV value of 48.75 for the genome, with individual chromosomes ranging from 46.08 to 51.67, reflecting the high accuracy of the assembly (Supplementary Table S7). Considering all these factors, the T2T *P. campanulata* genome presented here is of the highest reliability and quality.

Genome annotation analysis

Various prediction methods were employed to annotate repeat sequences in the *P. campanulata* T2T genome, yielding results for TE proteins and a combination of de novo and Repbase methods. After synthesizing the prediction results and eliminating redundancy, a total of 130.84 Mb of repeat sequences were identified, constituting 49.14% of the entire genome. This included 23.92% LTR retrotransposons, 15.47% DNA transposons, 4.63% long interspersed nuclear elements, 0.47% short interspersed nuclear elements, and 7.01% uncharacterized TEs (Fig. 2A, Supplementary Table S8). Employing a combination of de novo, homology-based, and transcriptome prediction methods, we identified 28,961 protein-coding genes in the *P. campanulata* genome (Table 2). The average lengths of transcripts, CDSs, exons, and introns were approximately 3,724 bp, 1,141 bp, 320 bp, and 523 bp, respectively, with an average of 5.02 exons per gene (Table 2). We analyzed the length distribution of genes, CDSs, exons, and introns among *C. serrulata*, *P. avium*, *P. mume*, *P. persica*, and *P. campanulata* v1.0. The exon and intron length distributions were consistent across species, with some variation in the gene and CDS length distributions, particularly in the *P. avium* genome (Fig. 2B). BUSCO analysis revealed that 98.2% (1,585 of 1,614) of the core conserved plant gene orthologs were fully detected, confirming the high-confidence annotation of these genes in *P. campanulata* (Supplementary Table S6). A significant majority (27,934; 96.45%) of the predicted protein-coding genes were successfully annotated by at least one gene function database, slightly higher than the 93.1% in the v1.0 assembly (Supplementary Table S9). Additionally, we identified a total of 2,414 noncoding RNAs, including 287 miRNAs, 668 tRNAs, 886 rRNAs, and 573 snRNAs, exceeding the numbers in the v1.0 assembly (Supplementary Table S10).

Table 2: Statistics of protein-coding genes annotation for *P. campanulata* genome.

Method	Software	Gene number	Average gene length (bp)	Average CDS length (bp)	Average exon per gene	Average exon length (bp)	Average intron length (bp)
Ab initio	GlimmmerHMM	36,212.00	4,849.50	911.42	3.76	242.55	1,428.07
Ab initio	AUGUSTUS	26,704.00	2,963.24	1,209.35	5.07	238.4	430.64
Homology	Exonerate	25,022.50	2,582.15	1,191.79	4.21	281.78	430.74
RNAseq	TransDecoder	26,816.00	4,481.45	999.95	5.63	397.82	484.19
Integration	Maker	27,457.00	4,111.52	1,130.70	5	286.72	668.32

Final set	Anno-self	28,961.00	3,724.42	1,140.73	5.02	320.28	523.14

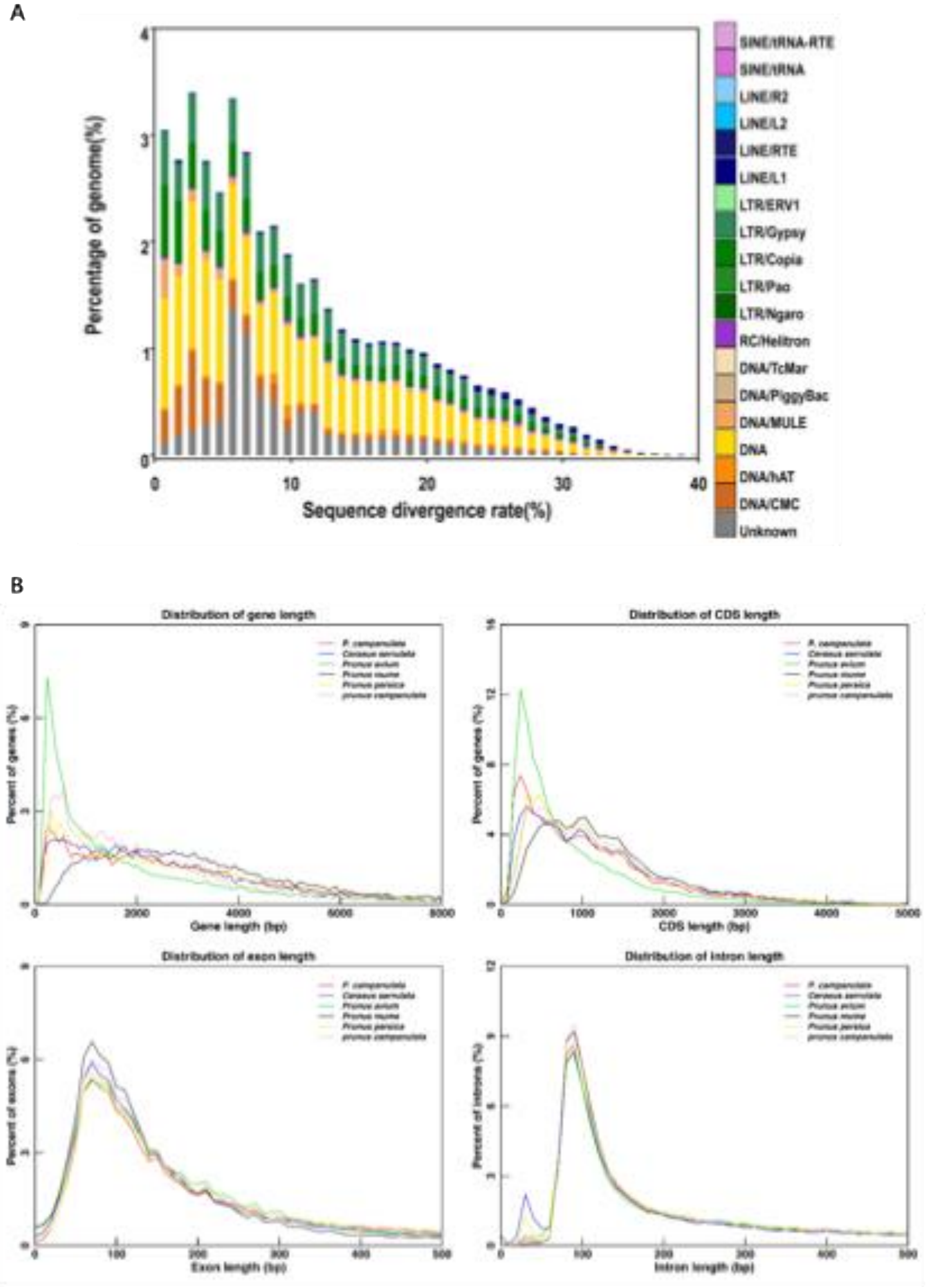

Figure 2: Genomic features of the *P. campanulata* genome. (A) Composition and divergence of transposable elements in the *P. campanulata* genome. (B) Distribution of gene length, coding sequence (CDS) length, exon length, and intron length among the assemblies of *P. campanulata* v2.0, *C. serrulata*, *P. avium*, *P. mume*, *P. persica*, and *P. campanulata* v1.0. The y-axis represents the percentage of genes, CDS, exons, and intron.

Genome-wide identification of variation

We conducted a comparative analysis with the v1.0 assembly, focusing on various sequences and SVs. A collinearity analysis revealed 270.82 Mb (97.5%) of syntenic regions between the v2.0 and v1.0 genomes (Fig. 3A). Within these syntenic regions, we identified 336,943 SNPs, of which 166,274 were distributed in gene regions and 170,669 in intergenic areas (Supplementary Table S11). Furthermore, we detected 107,521 InDels ranging from 2 to 50 bp, comprising 62,058 insertions and 45,463 deletions (Supplementary Table S11). A total of 1,413 SVs were identified, comprising 1,212 duplications, 163 translocations, and 38 inversions (Fig. 3B, Supplementary Fig. S2A, B, C). Structural annotation analysis indicated that most SVs were located 2 kb upstream and downstream of genes, in introns, and in intergenic regions, with a length distribution primarily centered around 1,001–2,000 bp and > 9,000 bp (Fig. 3C). Additionally, presence-absence variation (PAV) revealed 928 presence variants and 1,223 absence variants (Fig. 3B, Supplementary Fig. S2D). GO functional and KEGG pathway-enrichment analyses of these SV and PAV sequences showed significant enrichment in defense responses, including plant-pathogen interactions and secondary-metabolite biosynthesis (Supplementary Fig. S3). These findings underscore the importance of high-quality genome assembly in advancing plant research.

Newly annotated genes in *P. campanulata* T2T genome

By comparing gene models from the v2.0 assembly with those from v1.0, we identified 1,402 genes that were present in v2.0 but absent from v1.0, representing 4.9% of the protein-coding genes in v2.0 (Supplementary Fig. S4A, Supplementary Table S12). We conducted GO and KEGG pathway enrichment analyses to determine the functions of these newly annotated genes. The GO annotation results showed significant enrichment of genes involved in defense response within the biological process category, integral components of membranes within the cellular component category, and nucleic-acid binding and ATP binding within the molecular function category (Supplementary Fig. S4B). The KEGG annotations indicated that these genes were associated with processes detailed in the global and overview maps (Supplementary Fig. S4C) and with transcription.

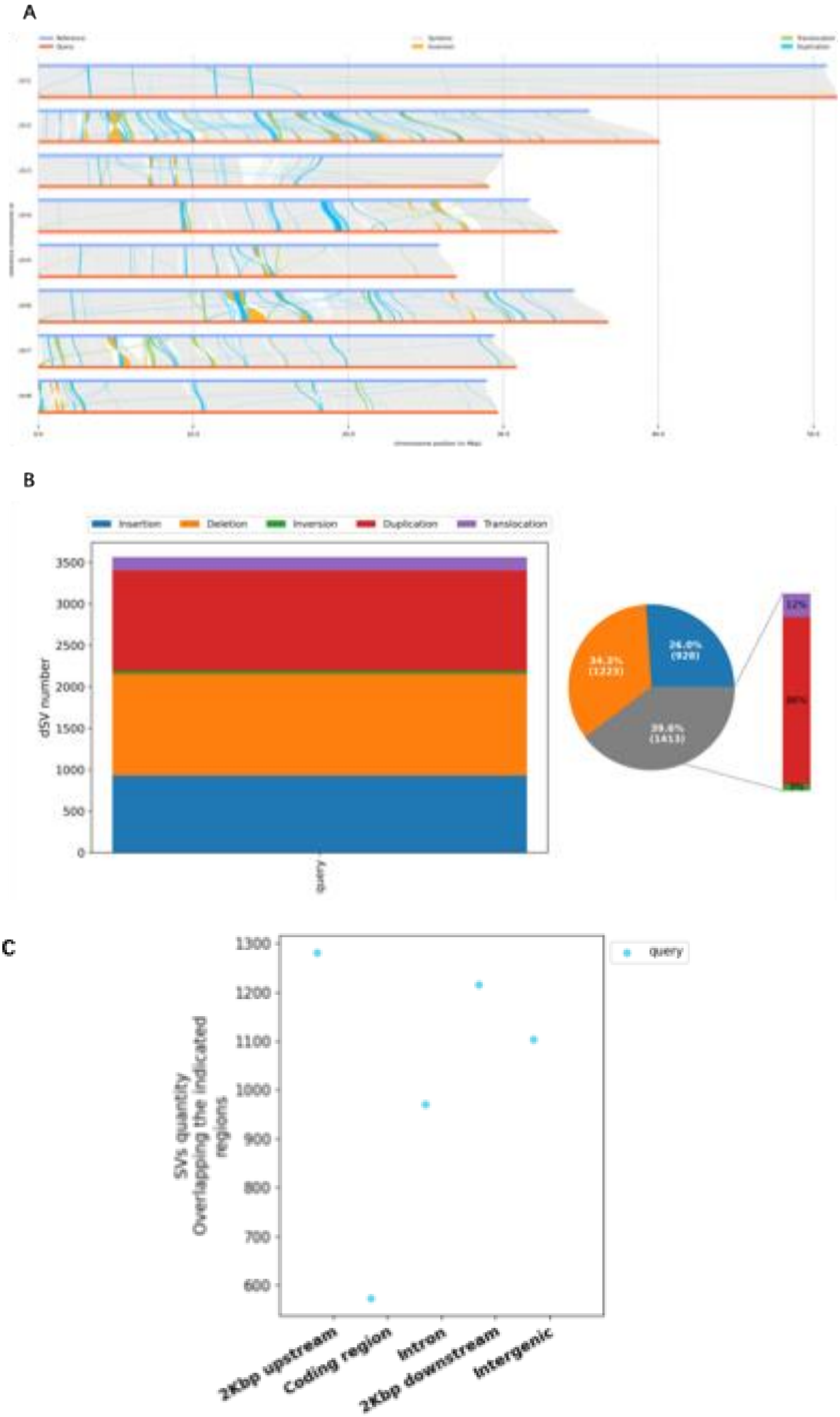
A
B
Insertion
Deletion
Inversion
Duplication
Translocation
dSV number
3500
3000
2500
2000
1500
1000
500
0
query
26.0%
(928)
34.3%
(1223)
39.6%
(1413)
12%
C
SVs quantity
Overlapping the indicated
regions
1300
1200
1100
1000
900
800
700
600
query
2Kbp upstream
Coding region
Intron
2Kbp downstream
Intergenic

Figure 3: Structural variations analysis between v2.0 and v1.0 *P. campanulata* genomes. (A) Collinearity between v2.0 and v1.0 *P. campanulata* genomes, with v2.0 as the reference. The gray lines show the syntenic regions between v2.0 and v1.0. Non-syntenic regions represent gaps in the v1.0 assembly. The orange, green, and bluish-green lines represent structural variations, specifically inversion, translocation, and duplication, respectively. (B) Number and proportion distribution of each type of structural variations. (C) Distribution of structural variations counts across different regions of the genome.

Telomere and centromere characteristics

Telomeres are essential conserved structures in plant genomes that differentiate natural chromosome ends from double-stranded breaks in DNA, thereby protecting the chromosome ends from degradation or end-to-end fusion with other chromosomes [96]. Typically, they are tandemly arranged minisatellites, following the formula (TxAyGz)n [74]. Utilizing telomere repeats as queries, we successfully identified 13 telomeres located at the ends of the eight pseudochromosomes (Table 3). Notably, chromosomes 4, 5, and 7 each had a telomere at only one end. The number of motif repeats ranged from a minimum of 158 to a maximum of 612 (Table 3). To predict potential centromere regions of the *P. campanulata* chromosomes, we utilized short tandem repeats, integrating these data with Hi-C interaction heat maps, large blank regions, areas of low gene density, and regions with high LTR/Gypsy density (Fig. 4). This approach successfully identified a presumptive centromere for each chromosome, with lengths ranging from 1.98 to 2.99 Mb (Table 3). However, verifying the actual locations of these centromeres will require further research, for example using FISH and ChIP-seq methods.

Table 3: Telomeres and centromeres in P. campanulata chromosomes.

Chromosome	Telomeres		Centromeres		
	Upstream	Downstream	Star	End	Length
Chr1	332	536	26000366	27989631	1989265
Chr2	204	330	22001493	23999214	1997721
Chr3	390	541	15001219	16998339	1997120
Chr4	0	248	4000087	5998911	1998824
Chr5	0	565	6002969	7994235	1991266
Chr6	199	306	15000384	17999009	2998625
Chr7	240	0	22503972	24999143	2495171
Chr8	158	612	18001542	19995827	1994285

Comparative genomic analysis

To examine the evolutionary dynamics of the flowering cherry genome, we conducted a comparative genomic analysis between the *P. campanulata* genome and those of 13 other species to identify homologous genes, performed gene family clustering analysis, and assessed the distribution of

single-copy and multiple-copy genes. A total of 74,894 orthologous gene families comprising 504,527 genes were detected across all species, with 7,893 gene families (encompassing 189,790 genes) shared by all (Fig. 5A, Supplementary Table S13). In addition, 182 single-copy gene families were identified. The *P. campanulata* v2.0 genome contained 1,198 unique gene families, including 1,446 unique paralogs (Supplementary Tables S13 and S14). GO enrichment analysis revealed that these gene families were predominantly involved in hexosyltransferase activity, fucose metabolic processes, and histidine biosynthesis (Supplementary Fig. S5A). KEGG pathway analysis indicated significant involvement in protein export, fructose and mannose metabolism, and nucleotide excision repair, among others (Supplementary Fig. S5B). Comparative analysis with the three closely related *Prunus* species *P. avium* [18], *C. serrulata* [22], and *P. persica* [51] showed that 13,844 gene families were shared among these species, while 1,636 gene families were unique to *P. campanulata* (Fig. 5B). These findings offer significant insights into the genetic uniqueness and evolutionary trajectory of the flowering cherry genome.

A phylogenetic tree was constructed using 177 single-copy orthologous genes from 14 species, with *A. thaliana* [76] serving as the outgroup. The resulting ML tree showed that *P. campanulata* forms a monophyletic clade with *P. yedoensis* [20], *C.* × *yedoensis* [21], *C. serrulata* [22], and *P. avium* [18], collectively classified under the subgenus *Cerasus* (Supplementary Fig. S6). This clade is sister to the subgenus *Prunus* clade that includes *P. salicina* [77], *P. mume* [50], and *P. persica* [51]. Phylogenetic analysis and fossil calibration indicated that *P. campanulata* and *P. avium* diverged approximately 23.4 Mya, with a 95% highest probability density (HPD) of 12.9–36.6 Mya (Supplementary Fig. S6, Supplementary Table S15). The divergence between the subgenus *Cerasus* and the subgenus *Prunus* occurred around 30.5 Mya, with a 95% HPD of 17.5–46.1 Mya. Employing the likelihood model in CAFE v3.1 with default parameters [90], we identified 377 gene family expansions and 1,272 gene family contractions affecting 1,543 and 1,473 genes, respectively (Fig. 5C). GO functional enrichment and KEGG pathway analyses revealed that the expanded gene families were primarily associated with pentose and glucuronate interconversions, fatty acid degradation, and tyrosine metabolism, whereas the contracted gene families were predominantly involved in plant-pathogen interactions, homologous recombination, and glutathione metabolism (Supplementary Fig. S7). WGD events in the *P. campanulata* genome were estimated by analyzing the synonymous mutation rates of homologous genes among *P. campanulata*, *P. avium*, *C. serrulata*, *P. persica*, and *M. domestica* [78], based on their orthologous gene pairs. The analysis revealed distinct peaks at 4DTv values of approximately 0.02 and 0.55 on the map (Supplementary Fig. S8A). The peak at approximately 0.02 4DTv highlighted divergence events between *P. campanulata* and *P. avium*, *P. campanulata* and *P. persica*, as well as *P. campanulata* and *P. mume*. The second peak at approximately 0.55 4DTv suggested a whole-genome or large-fragment duplication event in the common ancestor of these four species. Collinearity analysis between *P. campanulata* and *P. avium*, as well as between *P. campanulata* and *C. serrulata*, was conducted, showing an overall syntenic depth ratio of 1:1 for both comparisons (Fig. 5D, Supplementary Fig. S8B). This indicates that neither *P. campanulata* nor these related species experienced WGD events.

The study of positive selection of genes in plants is crucial to understanding their adaptive evolution. In this study, treating *P. campanulata* as the foreground branch and *P. avium*, *C. serrulata*, and *P. persica* as background branches, we identified several genes as candidates for positive

selection, genes that may have contributed to the species' evolutionary adaptation (Supplementary Table S16).

Conclusion

The T2T genomic assembly of *P. campanulata* 'Lianmeiren' v2.0 represents a significant advance in plant genomics, offering the first fully resolved, gapless genome for this species. In this study, we achieved a highly contiguous assembly with a contig N50 of 31.6 Mb and a total genome size of 266.23 Mb. The complete resolution of all eight pseudochromosomes, including the identification of 13 telomeres and prediction of centromere regions, provides unprecedented insights into the structural complexity of the *Prunus* genome. As the first complete T2T genome for a cherry species, this work marks a pivotal step toward understanding the genomic basis of important agronomic traits, ultimately contributing to the improvement of cherry cultivars and the broader field of plant genomics.

Abbreviations

BLAST: Basic Local Alignment Search Tool; bp: base pairs; BUSCO: Benchmarking Universal Single-Copy Orthologs; CDS: coding sequence; ChIP-seq: Chromatin Immunoprecipitation Sequencing; CTAB: cetyltrimethylammonium bromide; FISH: fluorescence in situ hybridization; Gb: gigabase pairs; GO: Gene Ontology; Hi-C: High-Throughput Chromosome Conformation Capture; HPD: highest posterior density; InDel: insertion-deletion; KEGG: Kyoto Encyclopedia of Genes and Genomes; Kb: kilobase pairs; KOG: EuKaryotic Orthologous Groups; LINE: long interspersed nuclear element; LRT: likelihood ratio test; LTR: long terminal repeat; Mb: megabase pairs; miRNA: micro RNA; ML: maximum likelihood; Mya: million years ago; Nr: NCBI's nonredundant database; ONT: Oxford Nanopore Technologies; PacBio HiFi: Pacific Biosciences high fidelity; PAV: presence and absence variation; rRNA: ribosomal RNA; RNA-seq: RNA sequencing; SDS: sodium dodecyl sulfate; SINE: short interspersed nuclear element; snRNA: small nuclear RNA; SNP: single nucleotide polymorphisms; SNR: signal-to-noise ratio; SV: structural variation; T2T: telomere to telomere; TE: transposable element; tRNA: transfer RNA; TRF: tandem repeat; WGD: whole-genome duplication; 4DTv: 4-fold degenerate synonymous sites of the third codons.

Additional Files

Supplementary Fig. S1. K-mer analysis of *P. campanulata* with GenomeScope (K = 19). Len, estimated total genome length; Uniq, unique portion of the genome (not repetitive); Aa, homozygosity rate; Ab, heterozygosity rate; Kcov, k-mer coverage for the heterozygous bases; Err, error rate; Dup, duplication rate; P, peak number.

Supplementary Fig. S2. Distribution of number and length of SVs. (A) Duplication. (B)

Translocation. (C) Inversion. (D) PAV.

Supplementary Fig. S3. GO and KEGG enrichment analysis of the SV and PAV genes. (A) Significantly enriched GO terms of the SV genes. (B) KEGG pathway enrichment of the SV genes. (C) Significantly enriched GO terms of the PAV genes. (D) KEGG pathway enrichment of the PAV genes.

Supplementary Fig. S4. Prediction and annotation of new genes in *P. campanulata* v2.0 genome. (A) Venn diagram of new gene prediction using de-novo, homology-based, and RNA-seq-based strategies. (B) GO analysis of new gene set, including biological process, cellular component, and molecular function. (C) KEGG pathway analysis of new gene set.

Supplementary Fig. S5. GO and KEGG pathway enrichment analysis of unique gene families between *P. campanulata* and the other 13 angiosperm species. (A) Significantly enriched GO terms of unique gene families. (B) KEGG pathway enrichment of unique gene families.

Supplementary Fig. S6. Phylogenetic tree and divergence time estimation. The numbers outside the square brackets indicate the average divergence time, and the numbers inside the square brackets indicate the 95% confidence interval of divergence time.

Supplementary Fig. S7. GO and KEGG pathway enrichment analysis of expansion and contraction gene families in *P. campanulata* v2.0 genome. (A) GO enrichment for expansion gene families. (B) KEGG pathway enrichment for expansion gene families. (C) GO enrichment for contraction gene families. (D) KEGG pathway enrichment for contraction gene families.

Supplementary Fig. S8. (A) Ka/Ks distribution of each pair of the five species genomes. Pcam: *P. campanulata*; Pavi: *P. avium*; Cser: *C. serrulata*; Pper: *P. persica*; Mdom: *M. domestica*. (B) Ratio of syntenic depth between *P. campanulata* and *C. serrulata*, and *P. avium*.

Supplementary Table S1. Summary of sequencing data of *P. campanulata* assembly.

Supplementary Table S2. Statistics and assessment of different assembly strategies.

Supplementary Table S3. Gap region and length in *P. campanulata* assembly.

Supplementary Table S4. The length and contig number of chromosomes in *P. campanulata* genome.

Supplementary Table S5. Assessment of *P. campanulata* assembly contiguity.

Supplementary Table S6. BUSCOs analysis of *P. campanulata* genome completeness.

Supplementary Table S7. QV analysis of *P. campanulata* genome accuracy.

Supplementary Table S8. Summary statistics of repetitive sequences in *P. campanulata* v2.0 genome.

Supplementary Table S9. Statistics of gene function annotation.

Supplementary Table S10. Statistics of noncoding RNAs in *P. campanulata* genome.

Supplementary Table S11. Statistics of SNP and InDel in *P. campanulata* genome.

Supplementary Table S12. Functional annotation of new genes.

Supplementary Table S13. Statistics of gene families of *P. campanulata* and the other 13 angiosperm species.

Supplementary Table S14. Statistics of orthologs genes between *P. campanulata* and the other 13 angiosperm species.

Supplementary Table S15. Fossil calibration points used to calibrate the phylogenetic tree.

Supplementary Table S16. GO annotation of genes under positive selection.

Authors' Contributions

Conceptualization and Supervision: D.J. and X.L. Methodology: X.S. and D.J. Software: X.S. and Y.L. Investigation: Q.Z. and F.Z. Writing—original draft preparation: X.S. and D.J. Writing—review and editing: X.S. and Y.L. Visualization: W.Z. and Q.Z. Funding acquisition: F.Z, X.S. and X.L. All authors have read and agreed to the published version of the manuscript.

Funding

This research was funded by the Special Support Funds of Zhejiang for Scientific Research Institutes, grant number 2023F1068-2. The research was supported by the Zhejiang Science and Technology Major Program on Agricultural New Variety Breeding, grant number 2021C02071-4 and the National Natural Science Foundation of China, grant number 32101585.

Data Availability

All raw sequencing data presented in this study have been deposited at NCBI (https://www.ncbi.nlm.nih.gov/) under BioProject accession number PRJNAXX. The genome assembly and annotation data are available at XX.

Competing Interests

All authors declare that they have no competing interests.

Acknowledgement

The authors thank Qiang Ou from Fujian Jinxiangyun Agricultural Development Co., Ltd. for providing the *P. campanulata* 'Lianmeiren' materials.

References

1. Shi S, Li J, Sun J, et al. Phylogeny and classification of *Prunus sensu lato* (Rosaceae). Journal of Integrative Plant Biology 2013;55:1069–79. https://doi.org/10.1111/jipb.12095.
2. Chin S-W, Shaw J, Haberle R, et al. Diversification of almonds, peaches, plums and c herries – Molecular systematics and biogeographic history of *Prunus* (Rosaceae). Molecu lar Phylogenetics and Evolution 2014;76:34–48. https://doi.org/10.1016/j.ympev.2014.02.02 4.
3. Shen X, Zong W, Li Y, et al. Evolution of cherries (*Prunus* Subgenus *Cerasus*) based on chloroplast genomes. International Journal of Molecular Sciences 2023;24:15612. http

s://doi.org/10.3390/ijms242115612.
4. Jiang D, Li X, Li Y, et al. Chromosome-level assembly of flowering cherry (*Prunus campanulata*) provides insight into anthocyanin accumulation. Genes 2023;14:389. https://doi.org/10.3390/genes14020389.
5. Nie C, Zhang Y, Zhang X, et al. Genome assembly, resequencing and genome-wide association analyses provide novel insights into the origin, evolution and flower colour variations of flowering cherry. The Plant Journal 2023:tpj.16151. https://doi.org/10.1111/tpj.16151.
6. Kato S, Matsumoto A, Yoshimura K, et al. Origins of japanese flowering cherry (*Prunus* subgenus *Cerasus*) cultivars revealed using nuclear SSR markers. Tree Genetics & Genomes 2014;10:477–87. https://doi.org/10.1007/s11295-014-0697-1.
7. Kanazawa Y, Kameyama Y, JingXiu L, et al. Genetic relationship between early-flowering cherry cultivars and regional populations of *Prunus campanulata*. Horticultural Research (Japan) 2016;15:129–38. https://doi.org/10.2503/hrj.15.129.
8. Guo Y, Kramer M, Pooler M. Screening ornamental cherry (*Prunus*) taxa for resistance to infection by Blumeriella jaapii. HortScience 2018;53:200–3. https://doi.org/10.21273/HORTSCI12563-17.
9. Garg V, Bohra A, Mascher M, et al. Unlocking plant genetics with telomere-to-telomere genome assemblies. Nat Genet 2024:1-12. https://doi.org/10.1038/s41588-024-01830-7.
10. Nurk S, Koren S, Rhie A, et al. The complete sequence of a human genome. Science 2022;376:44–53. https://doi.org/10.1126/science.abj6987.
11. Song J-M, Xie W-Z, Wang S, et al. Two gap-free reference genomes and a global view of the centromere architecture in rice. Molecular Plant 2021;14:1757–67. https://doi.org/10.1016/j.molp.2021.06.018.
12. Naish M, Alonge M, Wlodzimierz P, et al. The genetic and epigenetic landscape of the *Arabidopsis* centromeres. Science 2021;374:eabi7489. https://doi.org/10.1126/science.abi7489.
13. Chen J, Wang Z, Tan K, et al. A complete telomere-to-telomere assembly of the maize genome. Nat Genet 2023;55:1221–31. https://doi.org/10.1038/s41588-023-01419-6.
14. Shi X, Cao S, Wang X, et al. The complete reference genome for grapevine (*Vitis vinifera* L.) genetics and breeding. Horticulture Research 2023;10:uhad061. https://doi.org/10.1093/hr/uhad061.
15. Wang T, Wang B, Hua X, et al. A complete gap-free diploid genome in *Saccharum* complex and the genomic footprints of evolution in the highly polyploid *Saccharum* genus. Nat Plants 2023;9:554–571. https://doi.org/10.1038/s41477-023-01378-0.
16. Yu G, Matny O, Champouret N, et al. *Aegilops sharonensis* genome-assisted identification of stem rust resistance gene *Sr62*. Nat Commun 2022;13:1607. https://doi.org/10.1038/s41467-022-29132-8.
17. Zhang C, Xie L, Yu H, et al. The T2T genome assembly of soybean cultivar ZH13 and its epigenetic landscapes. Molecular Plant 2023;16:1715–8. https://doi.org/10.1016/j.molp.2023.10.003.
18. Shirasawa K, Isuzugawa K, Ikenaga M, et al. The genome sequence of sweet cherry (*Prunus avium*) for use in genomics-assisted breeding. DNA Research 2017;24:499–508. https://doi.org/10.1093/dnares/dsx020.

19. Wang J, Liu W, Zhu D, et al. Chromosome-scale genome assembly of sweet cherry (*Prunus avium* L.) cv. Tieton obtained using long-read and Hi-C sequencing. Hortic Res 2020;7:1–11. https://doi.org/10.1038/s41438-020-00343-8.
20. Baek S, Choi K, Kim G-B, et al. Draft genome sequence of wild *Prunus yedoensis* reveals massive inter-specific hybridization between sympatric flowering cherries. Genome Biol 2018;19:1–17. https://doi.org/10.1186/s13059-018-1497-y.
21. Shirasawa K, Esumi T, Hirakawa H, et al. Phased genome sequence of an interspecific hybrid flowering cherry, 'Somei-Yoshino' (*Cerasus* × *yedoensis*). DNA Research 2019;26:379–89. https://doi.org/10.1093/dnares/dsz016.
22. Yi X-G, Yu X-Q, Chen J, et al. The genome of Chinese flowering cherry (*Cerasus serrulata*) provides new insights into *Cerasus* species. Hortic Res 2020;7:165. https://doi.org/10.1038/s41438-020-00382-1.
23. Shirasawa K, Itai A, Isobe S. Genome sequencing and analysis of two early-flowering cherry (*Cerasus* × *kanzakura*) varieties, 'Kawazu-zakura' and 'Atami-zakura.' DNA Research 2021;28:dsab026. https://doi.org/10.1093/dnares/dsab026.
24. Wöhner TW, Emeriewen OF, Wittenberg AHJ, et al. The draft chromosome-level genome assembly of tetraploid ground cherry (*Prunus fruticosa* Pall.) from long reads. Genomics 2021;113:4173–83. https://doi.org/10.1016/j.ygeno.2021.11.002.
25. Jiu S, Chen B, Dong X, et al. Chromosome-scale genome assembly of *Prunus pusilliflora* provides novel insights into genome evolution, disease resistance, and dormancy release in *Cerasus* L. Horticulture Research 2023;10:uhad062. https://doi.org/10.1093/hr/uhad062.
26. Goeckeritz CZ, Rhoades KE, Childs KL, et al. Genome of tetraploid sour cherry (*Prunus cerasus* L.) "Montmorency" identifies three distinct ancestral *Prunus* genomes. Hortic Res 2023;10:uhad097. https://doi.org/10.1093/hr/uhad097.
27. Jiu S, Manzoor MA, Chen B, et al. Chromosome-level genome assembly provides insights into the genetic diversity, evolution, and flower development of *Prunus conradinae*. Mol Horticulture 2024;4:25. https://doi.org/10.1186/s43897-024-00101-7.
28. Chen S, Zhou Y, Chen Y, et al. fastp: an ultra-fast all-in-one FASTQ preprocessor. Bioinformatics 2018;34:i884–90. https://doi.org/10.1093/bioinformatics/bty560.
29. Wingett S, Ewels P, Furlan-Magaril M, et al. HiCUP: pipeline for mapping and processing Hi-C data. F1000Res 2015;4:1310. https://doi.org/10.12688/f1000research.7334.1.
30. Hu J, Wang Z, Sun Z, et al. NextDenovo: an efficient error correction and accurate assembly tool for noisy long reads. Genome Biol 2024;25:1–19. https://doi.org/10.1186/s13059-024-03252-4.
31. Walker BJ, Abeel T, Shea T, et al. Pilon: an integrated tool for comprehensive microbial variant detection and genome assembly improvement. PloS one 2014;9:e112963. https://doi.org/10.1371/journal.pone.0112963.
32. Cheng H, Concepcion GT, Feng X, et al. Haplotype-resolved de novo assembly using phased assembly graphs with hifiasm. Nat Methods 2021;18:170–5. https://doi.org/10.1038/s41592-020-01056-5.
33. Li H. Minimap2: pairwise alignment for nucleotide sequences. Bioinformatics 2018;34:3094–100. https://doi.org/10.1093/bioinformatics/bty191.
34. O'Leary NA, Wright MW, Brister JR, et al. Reference sequence (RefSeq) database at

NCBI: current status, taxonomic expansion, and functional annotation. Nucleic Acids Research 2016;44:D733–45. https://doi.org/10.1093/nar/gkv1189.
35. Zhang X, Zhang S, Zhao Q, et al. Assembly of allele-aware, chromosomal-scale autopolyploid genomes based on Hi-C data. Nat Plants 2019;5:833–45. https://doi.org/10.1038/s41477-019-0487-8.
36. Dudchenko O, Batra SS, Omer AD, et al. De novo assembly of the *Aedes aegypti* genome using Hi-C yields chromosome-length scaffolds. Science 2017;356:92–5. https://doi.org/10.1126/science.aal3327.
37. Durand NC, Shamim MS, Machol I, et al. Juicer provides a one-click system for analyzing loop-resolution Hi-C experiments. Cell systems 2016;3:95–8. https://doi.org/10.1016/j.cels.2016.07.002.
38. Durand NC, Robinson JT, Shamim MS, et al. Juicebox provides a visualization system for Hi-C contact maps with unlimited zoom. Cell systems 2016;3:99–101. https://doi.org/10.1016/j.cels.2015.07.012.
39. Wolff J, Rabbani L, Gilsbach R, et al. Galaxy HiCExplorer 3: a web server for reproducible Hi-C, capture Hi-C and single-cell Hi-C data analysis, quality control and visualization. Nucleic Acids Research 2020;48:W177–84. https://doi.org/10.1093/nar/gkaa220.
40. Jain C, Rhie A, Zhang H, et al. Weighted minimizer sampling improves long read mapping. Bioinformatics 2020;36:i111–8. https://doi.org/10.1093/bioinformatics/btaa435.
41. Kurtz S, Phillippy A, Delcher AL, et al. Versatile and open software for comparing large genomes. Genome Biol 2004;5:R12. https://doi.org/10.1186/gb-2004-5-2-r12.
42. Li H, Handsaker B, Wysoker A, et al. The sequence alignment/map format and SAMtools. Bioinformatics 2009;25:2078–9. https://doi.org/10.1093/bioinformatics/btp352.
43. Li H. Aligning sequence reads, clone sequences and assembly contigs with BWA-MEM. 2013, DOI: 10.48550/arXiv.1303.3997. https://doi.org/10.48550/arXiv.1303.3997.
44. Manni M, Berkeley MR, Seppey M, et al. BUSCO update: novel and streamlined workflows along with broader and deeper phylogenetic coverage for scoring of eukaryotic, prokaryotic, and viral genomes. Molecular Biology and Evolution 2021;38:4647–54. https://doi.org/10.1093/molbev/msab199.
45. Xu Z, Wang H. LTR_FINDER: an efficient tool for the prediction of full-length LTR retrotransposons. Nucleic Acids Research 2007;35:W265–8. https://doi.org/10.1093/nar/gkm286.
46. Ou S, Jiang N. LTR_retriever: a Highly accurate and sensitive program for identification of long terminal repeat retrotransposons. Plant Physiol 2018;176:1410–22. https://doi.org/10.1104/pp.17.01310.
47. Bao W, Kojima KK, Kohany O. Repbase Update, a database of repetitive elements in eukaryotic genomes. Mobile DNA 2015;6:1–6. https://doi.org/10.1186/s13100-015-0041-9.
48. Stanke M, Keller O, Gunduz I, et al. AUGUSTUS: *ab initio* prediction of alternative transcripts. Nucleic Acids Research 2006;34:W435–9. https://doi.org/10.1093/nar/gkl200.
49. Majoros WH, Pertea M, Salzberg SL. TigrScan and GlimmerHMM: two open source *ab initio* eukaryotic gene-finders. Bioinformatics 2004;20:2878–9. https://doi.org/10.1093/bioinformatics/bth315.
50. Zhang Q, Chen W, Sun L, et al. The genome of *Prunus mume*. Nat Commun 2012;3:1318. https://doi.org/10.1038/ncomms2290.

51. Verde I, Abbott AG, Scalabrin S, et al. The high-quality draft genome of peach (*Prunus persica*) identifies unique patterns of genetic diversity, domestication and genome evolution. Nat Genet 2013;45:487–94. https://doi.org/10.1038/ng.2586.
52. Slater GSC, Birney E. Automated generation of heuristics for biological sequence comparison. BMC Bioinformatics 2005;6:31. https://doi.org/10.1186/1471-2105-6-31.
53. Kim D, Paggi JM, Park C, et al. Graph-based genome alignment and genotyping with HISAT2 and HISAT-genotype. Nat Biotechnol 2019;37:907–15. https://doi.org/10.1038/s41587-019-0201-4.
54. Pertea M, Pertea GM, Antonescu CM, et al. StringTie enables improved reconstruction of a transcriptome from RNA-seq reads. Nat Biotechnol 2015;33:290–5. https://doi.org/10.1038/nbt.3122.
55. Holt C, Yandell M. MAKER2: an annotation pipeline and genome-database management tool for second-generation genome projects. BMC Bioinformatics 2011;12:491. https://doi.org/10.1186/1471-2105-12-491.
56. The UniProt Consortium. UniProt: the universal protein knowledgebase in 2021. Nucleic Acids Research 2021;49:D480–9. https://doi.org/10.1093/nar/gkaa1100.
57. Deng Y, Li J, Wu S, et al. Integrated nr database in protein annotation system and its localization. Computer Engineering 2006;32:71–2. https://doi.org/10.1109/INFOCOM.2006.241.
58. Ashburner M, Ball CA, Blake JA, et al. Gene Ontology: tool for the unification of biology. Nat Genet 2000;25:25–9. https://doi.org/10.1038/75556.
59. Koonin EV, Fedorova ND, Jackson JD, et al. A comprehensive evolutionary classification of proteins encoded in complete eukaryotic genomes. Genome Biol 2004;5:R7. https://doi.org/10.1186/gb-2004-5-2-r7.
60. Mistry J, Chuguransky S, Williams L, et al. Pfam: The protein families database in 2021. Nucleic Acids Research 2021;49:D412–9. https://doi.org/10.1093/nar/gkaa913.
61. Paysan-Lafosse T, Blum M, Chuguransky S, et al. InterPro in 2022. Nucleic Acids Research 2023;51:D418–27. https://doi.org/10.1093/nar/gkac993.
62. Kanehisa M, Goto S. KEGG: Kyoto Encyclopedia of Genes and Genomes. Nucleic Acids Research 2000;28(1):27-30. https://doi.org/10.1093/nar/28.1.27.
63. Buchfink B, Xie C, Huson DH. Fast and sensitive protein alignment using DIAMOND. Nat Methods 2015;12:59–60. https://doi.org/10.1038/nmeth.3176.
64. Bu D, Luo H, Huo P, et al. KOBAS-i: intelligent prioritization and exploratory visualization of biological functions for gene enrichment analysis. Nucleic Acids Research 2021;49:W317–25. https://doi.org/10.1093/nar/gkab447.
65. Jones P, Binns D, Chang H-Y, et al. InterProScan 5: genome-scale protein function classification. Bioinformatics 2014;30:1236–40. https://doi.org/10.1093/bioinformatics/btu031.
66. Eddy SR. Accelerated profile HMM searches. PLOS Computational Biology 2011;7:e1002195. https://doi.org/10.1371/journal.pcbi.1002195.
67. Chan PP, Lin BY, Mak AJ, et al. tRNAscan-SE 2.0: improved detection and functional classification of transfer RNA genes. Nucleic Acids Research 2021;49:9077–96. https://doi.org/10.1093/nar/gkab688.
68. Kalvari I, Nawrocki EP, Ontiveros-Palacios N, et al. Rfam 14: expanded coverage of

metagenomic, viral and microRNA families. Nucleic Acids Research 2021;49:D192–200. https://doi.org/10.1093/nar/gkaa1047.
69. Nawrocki EP, Eddy SR. Infernal 1.1: 100-fold faster RNA homology searches. Bioinfo rmatics 2013;29:2933–5. https://doi.org/10.1093/bioinformatics/btt509.
70. Goel M, Sun H, Jiao W-B, et al. SyRI: finding genomic rearrangements and local seq uence differences from whole-genome assemblies. Genome Biology 2019;20:277. https:// doi.org/10.1186/s13059-019-1911-0.
71. Xu L, Dong Z, Fang L, et al. OrthoVenn2: a web server for whole-genome comparis on and annotation of orthologous clusters across multiple species. Nucleic Acids Resear ch 2019;47:W52–8. https://doi.org/10.1093/nar/gkz333.
72. Wu T, Hu E, Xu S, et al. clusterProfiler 4.0: A universal enrichment tool for interpre ting omics data. Innovation 2021;2, DOI: 10.1016/j.xinn.2021.100141. https://doi.org/10.1 016/j.xinn.2021.100141.
73. Tang H, Krishnakumar V, Zeng X, et al. JCVI: A versatile toolkit for comparative ge nomics analysis. iMeta 2024;3:e211. https://doi.org/10.1002/imt2.211.
74. Podlevsky JD, Bley CJ, Omana RV, et al. The telomerase database. Nucleic Acids Re search 2008;36:D339–43. https://doi.org/10.1093/nar/gkm700.
75. Quinlan AR, Hall IM. BEDTools: a flexible suite of utilities for comparing genomic f eatures. Bioinformatics 2010;26:841–2. https://doi.org/10.1093/bioinformatics/btq033.
76. The Arabidopsis Genome Initiative. Analysis of the genome sequence of the flowering plant *Arabidopsis thaliana*. Nature 2000;408:796–815. https://doi.org/10.1038/35048692.
77. Liu C, Feng C, Peng W, et al. Chromosome-level draft genome of a diploid plum (*P runus salicina*). GigaScience 2020;9:giaa130. https://doi.org/10.1093/gigascience/giaa130.
78. Velasco R, Zharkikh A, Affourtit J, et al. The genome of the domesticated apple (*Ma lus* × *domestica* Borkh.). Nat Genet 2010;42:833–9. https://doi.org/10.1038/ng.654.
79. Gao Y, Yang Q, Yan X, et al. High-quality genome assembly of “Cuiguan” pear (*Pyr us pyrifolia*) as a reference genome for identifying regulatory genes and epigenetic mo difications responsible for bud dormancy. Horticulture Research 2021;8:197. https://doi.o rg/10.1038/s41438-021-00632-w.
80. Zhou Y, Xiong J, Shu Z, et al. The telomere-to-telomere genome of *Fragaria vesca* r eveals the genomic evolution of *Fragaria* and the origin of cultivated octoploid strawb erry. Horticulture Research 2023;10:uhad027. https://doi.org/10.1093/hr/uhad027.
81. Brůna T, Aryal R, Dudchenko O, et al. A chromosome-length genome assembly and a nnotation of blackberry (*Rubus argutus*, cv. ‘Hillquist’). 2022:2022.04.28.489789. https:// doi.org/10.1101/2022.04.28.489789.
82. Hibrand Saint-Oyant L, Ruttink T, Hamama L, et al. A high-quality genome sequence of *Rosa chinensis* to elucidate ornamental traits. Nature Plants 2018;4:473–84. https://do i.org/10.1038/s41477-018-0166-1.
83. Camacho C, Coulouris G, Avagyan V, et al. BLAST+: architecture and applications. BMC Bioinformatics 2009;10:421. https://doi.org/10.1186/1471-2105-10-421.
84. Emms DM, Kelly S. OrthoFinder: phylogenetic orthology inference for comparative ge nomics. Genome Biol 2019;20:238. https://doi.org/10.1186/s13059-019-1832-y.
85. Edgar RC. MUSCLE: multiple sequence alignment with high accuracy and high throug hput. Nucleic Acids Research 2004;32:1792–7. https://doi.org/10.1093/nar/gkh340.

86. Capella-Gutiérrez S, Silla-Martínez JM, Gabaldón T. trimAl: a tool for automated alignment trimming in large-scale phylogenetic analyses. Bioinformatics 2009;25:1972–3. https://doi.org/10.1093/bioinformatics/btp348.
87. Stamatakis A. RAxML version 8: a tool for phylogenetic analysis and post-analysis of large phylogenies. Bioinformatics 2014;30:1312–3. https://doi.org/10.1093/bioinformatics/btu033.
88. Yang Z. PAML 4: phylogenetic analysis by maximum likelihood. Molecular Biology and Evolution 2007;24:1586–91. https://doi.org/10.1093/molbev/msm088.
89. Kumar S, Suleski M, Craig JM, et al. TimeTree 5: an expanded resource for species divergence times. Molecular Biology and Evolution 2022;39:msac174. https://doi.org/10.1093/molbev/msac174.
90. Han MV, Thomas GWC, Lugo-Martinez J, et al. Estimating gene gain and loss rates in the presence of error in genome assembly and annotation using CAFE 3. Molecular Biology and Evolution 2013;30:1987–97. https://doi.org/10.1093/molbev/mst100.
91. Wang Y, Tang H, DeBarry JD, et al. MCScanX: a toolkit for detection and evolutionary analysis of gene synteny and collinearity. Nucleic Acids Res 2012;40:e49–e49. https://doi.org/10.1093/nar/gkr1293.
92. Wickham H. ggplot2. WIREs Computational Stats 2011;3:180–5. https://doi.org/10.1002/wics.147.
93. Katoh K, Standley DM. MAFFT multiple sequence alignment software version 7: improvements in performance and usability. Molecular Biology and Evolution 2013;30:772–80. https://doi.org/10.1093/molbev/mst010.
94. Suyama M, Torrents D, Bork P. PAL2NAL: robust conversion of protein sequence alignments into the corresponding codon alignments. Nucleic Acids Research 2006;34:W609–12. https://doi.org/10.1093/nar/gkl315.
95. Frith MC, Hamada M, Horton P. Parameters for accurate genome alignment. BMC Bioinformatics 2010;11:80. https://doi.org/10.1186/1471-2105-11-80.
96. Peska V, Garcia S. Origin, diversity, and evolution of telomere sequences in plants. Frontiers in Plant Science 2020;11. https://doi.org/10.3389/fpls.2020.00117.

Supplementary tables

Click here to access/download
Supplementary Material
Supplementary Tables.xlsx

Supplementary Fig S1

Click here to access/download
Supplementary Material
Figure S1 K-mer.pdf

Supplementary Fig S2

Click here to access/download
Supplementary Material
Figure S2 SV.pdf

Supplementary Fig S3

Click here to access/download
Supplementary Material
Figure S3 SV go and kegg.pdf

Supplementary Fig S4

Click here to access/download
Supplementary Material
Figure S4 New gene.pdf

Supplementary Fig S5

Click here to access/download
Supplementary Material
Figure S5 Unique gene families.pdf

Supplementary Fig S6

Click here to access/download
Supplementary Material
Figure S6 Phylogenetic tree and divertime.pdf

Supplementary Fig S7

Click here to access/download
Supplementary Material
Figure S7 Contraction and Expansion.pdf

Supplementary Fig S8

Click here to access/download
Supplementary Material
Figure S8 WGD.pdf

Cover letter Click here to access/download;Personal Cover;cover letter.docx

Dear Editors,

I am pleased to submit our manuscript titled "The telomere-to-telomere genome of flowering cherry (*Prunus campanulata*) reveals genomic evolution of the subgenus *Cerasus*" for consideration for publication in GigaScience. In this study, we present the first telomere-to-telomere (T2T) genome assembly of *P. campanulata* 'Lianmeiren', a significant step forward in the field of plant genomics, particularly for the *Prunus* genus.

The genome of *P. campanulata* 'Lianmeiren' has been partially sequenced before, but challenges in resolving highly repetitive regions and achieving a fully gapless assembly have persisted. Recent advances in sequencing technologies, including PacBio HiFi and Oxford Nanopore Technologies, have made it possible to overcome these challenges. Our study delivers a highly contiguous and accurate T2T assembly with a genome size of 266.23 Mb and a contig N50 of 31.6 Mb. The assembly exhibits exceptional completeness, with a BUSCO score of 98.9%, and high accuracy, with a QV of 48.75. We have successfully resolved all eight pseudochromosomes, identified 13 telomeres, and predicted centromere regions. Comparative analyses with the previous v1.0 assembly revealed significant structural variations and led to the annotation of 1,402 new genes. This T2T genome assembly provides a comprehensive and reliable reference that will significantly advance our understanding of the genetic architecture and evolutionary history of *P. campanulata*.

We believe that our findings will be of broad interest to the readers of GigaScience, particularly those involved in plant genomics, bioinformatics, and genome assembly. Our manuscript aligns well with the journal's focus on large-scale data generation and analysis, and the implications of our work are relevant to both fundamental research and applied horticulture.

We confirm that this manuscript is original, has not been published elsewhere, and is not under consideration by another journal. All authors have approved the manuscript and agreed to its submission to GigaScience. We have also complied with ethical standards and the data supporting our findings are available in public repositories, as detailed in the manuscript.

Thank you for considering our submission. We look forward to your feedback.

Sincerely Yours,

Corresponding author: Dongyue Jiang

Institute of Tree Breeding, Zhejiang Academy of Forestry,

Hangzhou 310023, China

Email: jiangdongyue@zjforestry.ac.cn

Phone: +86 571 8879 8027

Fax: +86 571 8879 8206
