# Supplementary material for: The telomere-to-telomere genome of flowering cherry (Prunus campanulata) reveals genomic evolution of the subgenus Cerasus
Source: Gigascience. 2025 Feb 21;14:giaf009. doi: 10.1093/gigascience/giaf009 (PMC11843098; doi:10.1093/gigascience/giaf009)
Supplement: giaf009_Response_to_Reviewer_Comments_Original_Submission [file giaf009_response_to_reviewer_comments_original_submission.pdf]

Author's Response To Reviewer Comments

Reviewer1
1. All uploaded data was results data from the analysis, preprocessed or data within calculation steps was not uploaded.

Response:
We appreciate the reviewer's observation and agree that providing preprocessed and intermediate data enhances the reproducibility and transparency of our study. To address this, we have now uploaded all raw sequencing data, preprocessed data, and intermediate files generated during the computational analysis to GigaDB (Revision folder) and NCBI SRA (ONT: SRR30780786; Hifi: SRR307807854; Illumina: SRR30780951; Hi-C: SRR21901709; Transcript: SRR21912487 ~ SRR21912493). The repository contains detailed metadata and a step-by-step guide describing how the raw data were processed and used in our analyses. The relevant accession numbers and links have been included in the revised manuscript under the "Data Availability" section.
Additionally, we have verified that all essential calculation steps are documented and supported by the corresponding data. This ensures that the analyses can be reproduced accurately by readers.
Please let us know if there are additional suggestions or specific types of data that need further clarification.

2. The manuscript starts with the initial structure given by the authors guidelines (Context and Methods). Both sections full fill the requirements. However, the results section and conclusion does not fit to the recommended structure. I would suggest to restructure some results parts into a clear Data validation and quality control (please assign it in that way) paragraph (T2T assembly of P. campanulata genome, Extensive evaluation of the T2T P. campanulata assembly, Genome annotation + Newly annotated genes in P. campanulata T2T genome, genome wide identification of variation, telomere and centromere characteristics, comparative genomic analysis). These parts are very detailed which is commendable, but sometime it is also to long. Maybe there is possibility to summarize some of the paragraphs into one and to refer more to supplemental material parts.

Response:
Thank you for your valuable feedback. We acknowledge your concern regarding the structure of the Results and Conclusion sections. However, as observed in numerous Data Note articles published in GigaScience, the structure of the Results in our manuscript is consistent with the flexible approach often adopted for this article type. This format allows for a concise presentation of findings and highlights the dataset's significance without strictly adhering to traditional research article structures.
To ensure clarity and to better address your suggestion, we have revised the manuscript to emphasize the potential for data reuse in the "Results" section, and deleted "Conclusion" section.

3. To my opinion, the potential to reuse the data is not clearly outlined and written in the data note. The authors should clearly address why the genome is a source of data and what potential reuses were given by that dataset. This should not only be in general, some examples from Prunus genetics would be necessary to explain the reader the potential reuse (example: in the genome 1400 new genes were identified - have these genes been investigated or found in other Prunus species? Are these genes a potent source or a gene that controls a trait of interest for breeders? Could this data be used in another future study - to generate a haplotype-resolved genome of P. campanulata?) - It is written that genes were explored but not in what context these explorations could be used in future research or practical application. For such an explanation, it is not necessary to investigate the genes itself, but it should be explained in the text which genes or traits might exist in other Prunus species and that the genome could hold potential resources for these applications.

Response:
We appreciate the reviewer's detailed suggestions and acknowledge the importance of clearly outlining the potential for data reuse, particularly in the context of Prunus genetics. To address these concerns, we have revised the relevant sections of the manuscript, incorporating specific examples and elaborating on the

potential applications of the P. campanulata genome.

Reviewer2
1. While haplotype resolution might be unnecessary if heterozygosity is low, it would be appropriate to specify how heterozygous regions were handled during the assembly process.

Response:
We appreciate the reviewer’s insightful comment regarding the handling of heterozygous regions during the assembly process. In our study, Prunus campanulata ‘Lianmeiren’ was estimated to have a low heterozygosity rate of 0.54%, making haplotype resolution less critical. However, to ensure the integrity and accuracy of the assembly, we specifically addressed heterozygous regions using established tools.
We have revised the Methods section of the manuscript to include this information, ensuring that the process for managing heterozygous regions is clearly documented. We hope this addresses the reviewer’s concern, and we remain open to additional suggestions.

2. The genome presented in this work is a substantial improvement over the previous P. campanulata assembly, but I haven't been able to access the assembly and annotation. Additionally it is unclear if they have been deposited and how they will be accessible after the paper publication.

Response:
Thank you for your observation regarding the accessibility of the assembly and annotation files. We have deposited the genome assembly and annotation files into the NCBI database, and the submission is currently under processing. Once the processing is complete, the dataset, including the raw sequencing data, final T2T genome assembly, and annotation files, will be publicly accessible.
In the revised manuscript, we have updated the “Data Availability” section to provide the accession numbers issued by NCBI. Additionally, we confirm that the data will be openly available upon publication of the paper to ensure reproducibility and support further research.
We apologize for any inconvenience caused by this delay and appreciate your understanding. If necessary, we are happy to share interim access to the data upon request during the review process.

3. In the methods section, under the paragraph "Plant material and sequencing," I would add a citation for the CTAB extraction method, even though it is a standard method in molecular biology laboratories. A reference is also needed when the SDS method is mentioned.

Response:
Thank you for your suggestion to include references for the DNA extraction methods mentioned in the “Plant material and sequencing” section. We agree that providing citations, even for widely used methods, enhances the rigor and traceability of the manuscript.
To address this, we have added appropriate references for both the CTAB and SDS extraction methods in the revised manuscript.

4. In the same paragraph, the name of the Nanopore kit for Ultra-long DNA sequencing is SQK-ULK114 and not SQK-LSK114.

Response:
We appreciate the reviewer’s attention to detail and for pointing out the error in the name of the Nanopore kit used for Ultra-long DNA sequencing. We have corrected this in the Methods section of the revised manuscript, changing “SQK-LSK114” to “SQK-ULK114” as per the reviewer’s suggestion.
Thank you for bringing this to our attention. This correction ensures the accuracy of the methodological details presented in the manuscript.

5. In the last line of the paragraph “Genome assembly and assessment,” the abbreviation “QV” is used but its meaning is not explained. From the context, it seems to be an estimate of assembly quality, but I would add a brief explanation of its meaning.

Response:
Thank you for pointing out the need to clarify the abbreviation “QV” in the “Genome assembly and

assessment” section. We agree that a brief explanation will improve the reader’s understanding.
In the revised manuscript, we have added an explanation of “QV” where it is first mentioned. Specifically, we now state that “QV” refers to the “quality value,” which is a logarithmic score representing the accuracy of the genome assembly. A higher QV indicates fewer errors per base and reflects a higher-quality assembly.
We appreciate your suggestion, as it enhances the clarity and accessibility of this section for readers unfamiliar with the term.

6. In the "Evolutionary analysis" section, 13 species are listed for comparison, but it is not entirely clear which version is used for some genomes; for example, for Arabidopsis and apple, the first published genome is cited, but these are certainly not the two most complete versions available now.

Response:
We appreciate the reviewer’s comment and agree that specifying the genome versions used for comparison is important for clarity and reproducibility.
For Arabidopsis thaliana, we used the well-established TAIR10.1 annotation, which remains the most reliable and widely used reference for functional and comparative genomics. Similarly, for Malus domestica, we utilized the Malus domestica v3.0.a1 genome that includes a comprehensive annotation file, enabling consistent comparisons with the other species in our analysis. While recent genome assemblies are available for some species, such as Prunus avium and Rosa chinensis, these newer assemblies lack accompanying high-quality annotation files, which are essential for the gene family clustering and evolutionary analyses conducted in this study.
We have updated the “Evolutionary analysis” section of the manuscript to specify the exact versions of the genomes and annotations used for all 13 species, along with citations for the corresponding references. We believe this approach ensures methodological transparency while maintaining the accuracy and consistency of the comparative analyses.
Thank you for raising this point, as it allowed us to clarify these details in the revised manuscript.

7. Also in the "Evolutionary analysis" section, the "chi2 program" is mentioned but a reference is missing.

Response:
Thank you for pointing out the need to include a reference for the “chi2 program” mentioned in the “Evolutionary analysis” section. In the revised manuscript, we have added a proper citation for the chi-squared test implementation used in the analysis. Specifically, we referenced the module provided in the PAML software package, which is widely used for likelihood ratio tests in phylogenetic and evolutionary studies.
We appreciate your attention to this detail, as it ensures proper attribution and enhances the methodological rigor of the manuscript.

8. The quality/resolution of the figures included in the main text is low, and it is difficult to see all the details, but this could be due to the PDF provided for review.

Response:
Thank you for bringing this to our attention. We apologize for any issues with the quality or resolution of the figures in the main text provided for review. This may have resulted from file compression during manuscript submission.
To address this, we have ensured that all figures are provided in high resolution (300 dpi or higher) in the revised submission. We believe this will resolve the issue and ensure that all details in the figures are easily visible.
Please let us know if further adjustments are needed.
