# Supplementary material for: The telomere-to-telomere genome of flowering cherry (Prunus campanulata) reveals genomic evolution of the subgenus Cerasus
Source: Gigascience. 2025 Feb 21;14:giaf009. doi: 10.1093/gigascience/giaf009 (PMC11843098; doi:10.1093/gigascience/giaf009)
Supplement: giaf009_Reviewer_1_Report_Original_Submission — ThomasWolfgangWÃ¶hner -- 10/27/2024 [file giaf009_reviewer_1_report_original_submission.pdf]

Reviewer Report

Title: The telomere-to-telomere genome of flowering cherry (Prunus campanulata) reveals genomic evolution of the subgenus Cerasus

Version: Original Submission **Date:** 10/27/2024

Reviewer name: Thomas Wolfgang WÃ¶hner

Reviewer Comments to Author:

Review GIGA-D-24-00376: The telomere-to-telomere genome of flowering cherry (Prunus campanulata) reveals genomic evolution of the subgenus Cerasus1) Are the methods appropriate to the aims of the study, are they well described, and are necessary controls included? - yes.2) Are the conclusions adequately supported by the data shown? - yes, but the structure of the manuscript is not appropriate in the context of the data note.3) Please indicate the quality of language in the manuscript. Does it require a heavy editing for language and clarity? - no.4) Are you able to assess all statistics in the manuscript, including the appropriateness of statistical tests used? - no, I did not check, however the possibility of assessment was given.Dear editor,after revising the manuscript I will give you the following recommendation: major revision.The work presented by Shen et al. is novel and state of the art technology/analysis was used to create a T2T pseudo-chromosome level genome sequence of Prunus campanulata. The manuscript is clearly written and no language improvements are necessary to my opinion. All uploaded data was results data from the analysis, preprocessed or data within calculation steps was not uploaded. Therefore a recalculation of the presented data is not really possible, but due to changing software versions and access to computational resources it is hard recalculate and control the calculations. To my opinion the calculations and methods sounds solid. The manuscript starts with the initial structure given by the authors guidelines (Context and Methods). Both sections full fill the requirements. However the results section and conclusion does not fit to the recommended structure. I would suggest to restructure some results parts into a clear Data validation and quality control (please assign it in that way) paragraph (T2T assembly of P. campanulata genome, Extensive evaluation of the T2T P. campanulata assembly, Genome annotation + Newly annotated genes in P. campanulata T2T genome, genome wide identification of variation, telomere and centromere characteristics, comparative genomic analysis). These parts are very detailed which is commendable, but sometime it is also to long. Maybe there is possibility to summarize some of the paragraphs into one and to refer more to supplemental material parts.To my opinion, the potential to reuse the data is not clearly outlined and written in the data note. The authors should clearly address why the genome is a source of data and what potential reuses were given by that dataset. This should not only be in general, some examples from Prunus genetics would be necessary to explain the reader the potential reuse (example: in the genome 1400 new genes were identified - have these genes been investigated or found in other Prunus species? Are these genes a potent source or a gene that controls a trait of interest for breeders?, could this data be used in another future study - to generate a haplotype resolved genome of P. campanulata?) - It is written that genes were explored but not in what context these explorations could be used in future research or practical application. For such an explanation, it is not necessary to

investigate the genes itself, but it should be explained in the text which genes or traits might exist in other Prunus species and the that the genome could hold potential resources for these application. Summarizing, these paragraph is missing and I strongly recommend the authors to add such a paragraph to the text. Moreover, the authors should check if the reference style fit to the guideline of Gigascience.author guidelines:https://academic.oup.com/gigascience/pages/data_note)Structure given by Gigascience:ContextMethodsData Validation and quality controlRe-use potential

Level of Interest

Please indicate how interesting you found the manuscript: Choose an item.

Quality of Written English

Please indicate the quality of language in the manuscript: Choose an item.

Declaration of Competing Interests

Please complete a declaration of competing interests, considering the following questions:

- Have you in the past five years received reimbursements, fees, funding, or salary from an organisation that may in any way gain or lose financially from the publication of this manuscript, either now or in the future?
- Do you hold any stocks or shares in an organisation that may in any way gain or lose financially from the publication of this manuscript, either now or in the future?
- Do you hold or are you currently applying for any patents relating to the content of the manuscript?
- Have you received reimbursements, fees, funding, or salary from an organization that holds or has applied for patents relating to the content of the manuscript?
- Do you have any other financial competing interests?
- Do you have any non-financial competing interests in relation to this paper?

If you can answer no to all of the above, write 'I declare that I have no competing interests' below. If your reply is yes to any, please give details below.

I declare that I have no competing interests.

I agree to the open peer review policy of the journal. I understand that my name will be included on my report to the authors and, if the manuscript is accepted for publication, my named report including any attachments I upload will be posted on the website along with the authors' responses. I agree for my report to be made available under an Open Access Creative Commons CC-BY license (http://creativecommons.org/licenses/by/4.0/). I understand that any comments which I do not wish to

be included in my named report can be included as confidential comments to the editors, which will not be published.

Choose an item.

To further support our reviewers, we have joined with Publons, where you can gain additional credit to further highlight your hard work (see: https://publons.com/journal/530/gigascience). On publication of this paper, your review will be automatically added to Publons, you can then choose whether or not to claim your Publons credit. I understand this statement.

Yes Choose an item.
