# Supplementary material for: The telomere-to-telomere genome of flowering cherry (Prunus campanulata) reveals genomic evolution of the subgenus Cerasus
Source: Gigascience. 2025 Feb 21;14:giaf009. doi: 10.1093/gigascience/giaf009 (PMC11843098; doi:10.1093/gigascience/giaf009)
Supplement: giaf009_Reviewer_2_Report_Original_Submission — DiegoMicheletti --11/8/2024 [file giaf009_reviewer_2_report_original_submission.pdf]

Reviewer Report

Title: The telomere-to-telomere genome of flowering cherry (Prunus campanulata) reveals genomic evolution of the subgenus Cerasus

Version: Original Submission **Date:** 11/8/2024

Reviewer name: Diego Micheletti

Reviewer Comments to Author:

In this article, Shen and colleagues present a T2T assembly of the P. campanulata, a spacies of flowering cherry, genome, although it is not clear whether the authors have resolved the two haplotypes. While haplotype resolution might be unnecessary if heterozygosity is low, it would be appropriate to specify how heterozygous regions were handled during the assembly process. The article is very well-written, especially the methods section that is always very detailed.The genome presented in this work is a substantial improvement over the previous P. campanulata assembly, but I haven't been able to access the assembly and annotation. Additionally it is unclear if they have been deposited and how they will be accessible after the paper publication.I have a few minor suggestions to slightly improve the article. In the methods section, under the paragraph "Plant material and sequencing," I would add a citation for the CTAB extraction method, even though it is a standard method in molecular biology laboratories. A reference is also needed when the SDS method is mentioned. In the same paragraph, the name of the Nanopore kit for Ultra-long DNA sequencing is SQK-ULK114 and not SQK-LSK114. In the last line of the paragraph "Genome assembly and assessment," the abbreviation "QV" is used but its meaning is not explained. From the context, it seems to be an estimate of assembly quality, but I would add a brief explanation of its meaning.In the "Evolutionary analysis" section, 13 species are listed for comparison, but it is not entirely clear which version is used for some genomes; for example, for Arabidopsis and apple, the first published genome is cited, but these are certainly not the two most complete versions available now. If the most recent version of the selected genomes was not used, the authors should justify their choice.Also in the "Evolutionary analysis" section, the "chi2 program" is mentioned but a reference is missing.The quality/resolution of the figures included in the main text is low, and it is difficult to see all the details, but this could be due to the PDF provided for review."

Level of Interest

Please indicate how interesting you found the manuscript: Choose an item.

Quality of Written English

Please indicate the quality of language in the manuscript: Choose an item.

Declaration of Competing Interests

Please complete a declaration of competing interests, considering the following questions:

- Have you in the past five years received reimbursements, fees, funding, or salary from an organisation that may in any way gain or lose financially from the publication of this manuscript, either now or in the future?
- Do you hold any stocks or shares in an organisation that may in any way gain or lose financially from the publication of this manuscript, either now or in the future?
- Do you hold or are you currently applying for any patents relating to the content of the manuscript?
- Have you received reimbursements, fees, funding, or salary from an organization that holds or has applied for patents relating to the content of the manuscript?
- Do you have any other financial competing interests?
- Do you have any non-financial competing interests in relation to this paper?

If you can answer no to all of the above, write 'I declare that I have no competing interests' below. If your reply is yes to any, please give details below.

I declare that I have no competing interests

I agree to the open peer review policy of the journal. I understand that my name will be included on my report to the authors and, if the manuscript is accepted for publication, my named report including any attachments I upload will be posted on the website along with the authors' responses. I agree for my report to be made available under an Open Access Creative Commons CC-BY license (http://creativecommons.org/licenses/by/4.0/). I understand that any comments which I do not wish to be included in my named report can be included as confidential comments to the editors, which will not be published.

Choose an item.

To further support our reviewers, we have joined with Publons, where you can gain additional credit to further highlight your hard work (see: https://publons.com/journal/530/gigascience). On publication of this paper, your review will be automatically added to Publons, you can then choose whether or not to claim your Publons credit. I understand this statement.

Yes Choose an item.
